# Enterococcal biofilm—A nidus for antibiotic resistance transfer?

**DOI:** 10.1111/jam.15441

**Published:** 2022-01-26

**Authors:** Michael Conwell, James S. G. Dooley, Patrick J. Naughton

**Affiliations:** ^1^ The Nutrition Innovation Centre for Food and Health (NICHE) School of Biomedical Sciences Ulster University Coleraine UK

**Keywords:** antibiotic resistance, biofilm, enterococci, horizontal gene transfer

## Abstract

Enterococci, which are on the WHO list of priority pathogens, are commonly encountered in hospital acquired infection and are becoming increasing significant due to the development of strains resistant to multiple antibiotics. Enterococci are also important microorganisms in the environment, and their presence is frequently used as an indicator of faecal pollution. Their success is related to their ability to survive within a broad range of habitats and the ease by which they acquire mobile genetic elements, including plasmids, from other bacteria. The enterococci are frequently present within a bacterial biofilm, which provides stability and protection to the bacterial population along with an opportunity for a variety of bacterial interactions. Enterococci can accept extrachromosomal DNA both from within its own species and from other bacterial species, and this is enhanced by the proximity of the donor and recipient strains. It is this exchange of genetic material that makes the role of biofilms such an important aspect of the success of enterococci. There remain many questions regarding the most suitable model systems to study enterococci in biofilms and regarding the transfer of genetic material including antibiotic resistance in these biofilms. This review focuses on some important aspects of biofilm in the context of horizontal gene transfer (HGT) in enterococci.

## INTRODUCTION

The continued increase in antibiotic resistance among human and animal pathogens is a threat to public health. Multiresistant organisms such as vancomycin resistant enterococci (VRE) have emerged as major threats to human health, particularly in healthcare settings worldwide (Faron et al., 2016; Hung et al., 2019; Uçkay et al., 2017) as well as in the environment (Huijbers et al., 2015), and *Enterococcus faecalis* (Efs) is an extremely common gut commensal of animals (including humans), (Lebreton et al., 2014). In the last decade, a fundamental reappraisal of how bacteria grow under environmental conditions has taken place (reviewed in detail by Haruta & Kanno, 2015). It is now clear that many bacteria exist as part of complex communities attached to surfaces, embedded in polymeric matrices of their own devising known as biofilm (reviewed in detail by Flemming et al., 2016). Biofilms were first identified in aquatic environments, such as rock surfaces in streams, but have now been recognized as major contributors to infection (Ch'ng et al., 2019; Høiby et al., 2015). Clinically, biofilms are found in a wide range of disease states, from indwelling medical devices and urinary tract infections to diabetic ulcers and the lungs of cystic fibrosis (CF) patients. Biofilms allow colonization of a variety of inanimate materials as well as forming directly on body surfaces thereby facilitating chronic infections such as post‐surgical infections, endocarditis, otitis media, etc. Their significant clinical impact has recently been comprehensively reviewed by Schulze et al. (2021). The regulation of the physiological processes of biofilms is poorly understood (O’Toole & Wong, 2016; Santos‐Beneit, 2015). Moreover, despite antibiotic resistance transfer mechanisms being described for enterococci growing in planktonic culture, our understanding of how efficiently these mechanisms function in biofilm is limited (Van Acker et al., 2014).

Various hypotheses have been put forward regarding what comprises a biofilm matrix and its cellular components, including the development of a multifaceted structure comprising adherent organisms (Dunny et al., 2014). Biofilm models of ‘development’ and biological function were initially based upon research carried out on *Pseudomonas aeruginosa* and *P*. *fluorescens* although the limitations of extrapolating these to Gram positive organisms have long been recognized (Monds & O’Toole, 2009). With regards to enterococci, models of development are still not fully understood, and an understanding of biological functions is just beginning to emerge (Gilmore et al., 2014; Kim et al., 2020). Biofilms have been shown to play a role in some enterococcal nosocomial infections, for example, providing a location for the attachment of a population of bacteria to a heart valve during endocarditis (O'Toole et al., 2000). The initial colonizing isolate produces anchoring sites through the release of DNA and polymeric substances, paving the way for later additions of new members using the DNA as an attachment site (Mohamed & Huang, 2007; O'Toole et al., 2000). The stepwise addition of new members to the microcolony biofilm means that late colonizers can consist of bacteria that could never form biofilm at the specific site due to issues with nutrient availability and oxygen saturation (Whitchurch et al., 2002). Barnes et al. (2017) who studied the colonization of Efs, noted that the rescue of mutant phenotypes by parent or other mutant strains within a pool had the potential to complicate the findings of biofilms studies in vivo. Moreover, rescue attempts in a cooperative environment may see the rise of cheaters that do not contribute as has been seen in other bacterial communities (Pollak et al., 2016).

**FIGURE 1:**
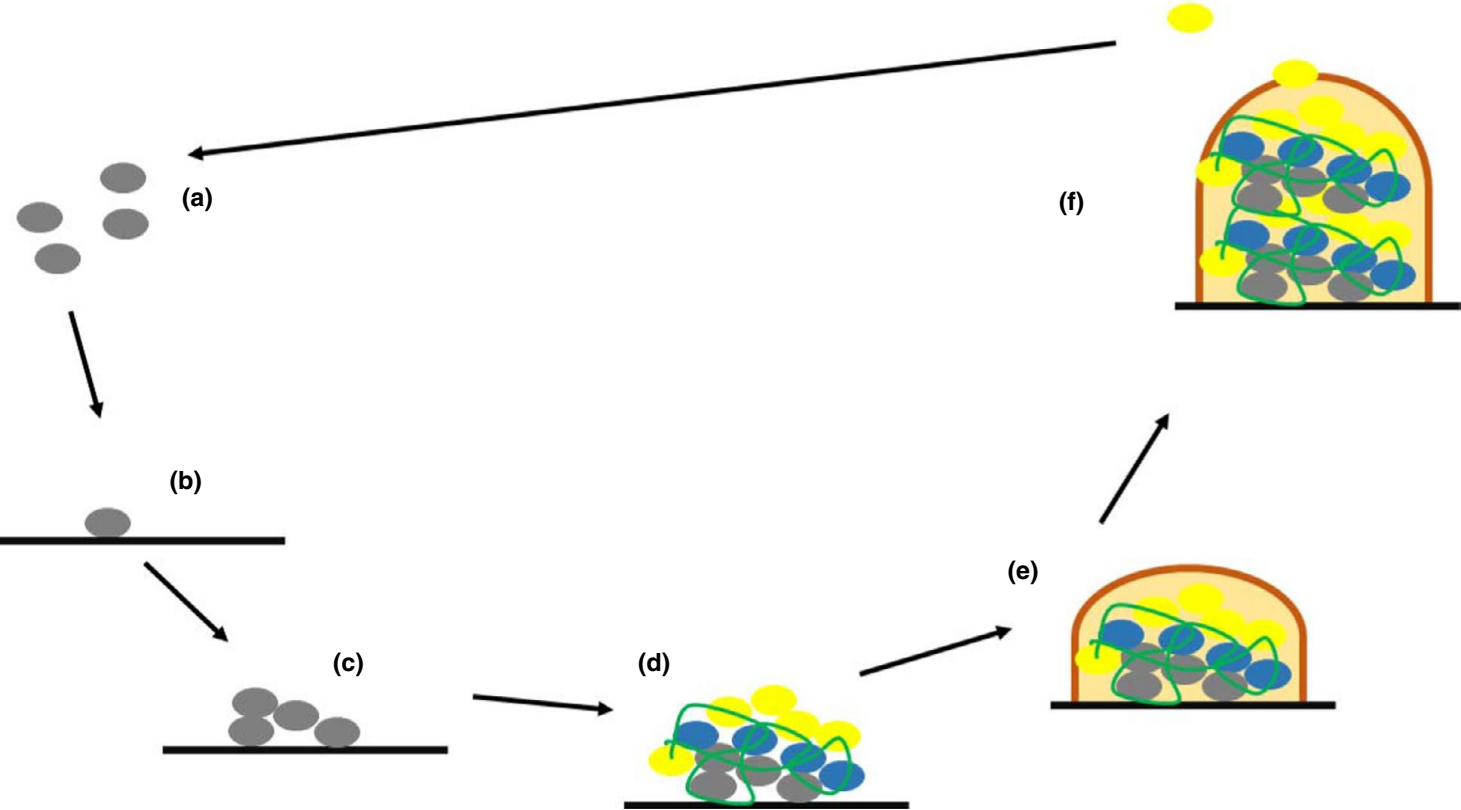
The biofilm development and maturation cycle of enterococci in a multispecies biofilm. Created using information from Dunny et al. (2014). (a) Planktonic enterococci (grey oval). (b) Irreversible binding of enterococci to abiotic substrate, rich in nutrients, iron, CO_2_, low osmolarity. (c) Production of ESP, gelatinase, attachment/aggregation of clones though quorum sensing. (d) Secretion of eDNA (green line). Modulation of environment allows attachment of other bacteria (blue and yellow ovals). (e) Multi‐organism secretion of polysaccharides and exopolymers (brown chord). (f) Maturation of biofilm and bacterial communal release

Having several attachment sites in a given biofilm microcolony (biotic or abiotic surface, EPS or even directly to proteins on bacterial cell surfaces) may allow more pathogenic species of bacteria to enter the biofilm, creating continual sites for chronic, systemic infection (Gill et al., 2005; O'Toole et al., 2000; Vuong et al., 2004). Mature biofilms are usually harder to eradicate due to increased surface area of attachment, formation characteristics and the polymicrobial nature of the biofilm itself (Boles et al., 2004; Rochex et al., 2008). Where enterococci are concerned, the expression of the enterococcal surface protein (ESP), a cell wall‐associated protein, has shown improved adhesion and therefore biofilm formation (Toledo‐Arana et al., 2001). Studies by Kristich et al. (2004) and Tendolkar et al. (2004) concluded that ESP must act in coordination with various factors involved in enterococcal biofilm formation, and its presence can improve biofilm formation. Hence, enterococcal biofilm is now described as multifactorial in nature (Dunny et al., 2014; Garg et al., 2017). Additionally, enterococcal gelatinase contributes to biofilm during infection by hydrolysing host tissues (collagen, fibrinogen and fibrin) into derivatives (gelatin, various peptides and amino acids) and is recognized as a key virulence factor (Hancock & Perego, 2004). Mediated through the *Fsr* quorum response, gelatinase provides both nutrients and anchoring sites for the development of biofilm through aiding in the production of aggregation substance (Fisher & Phillips, 2009; Thurlow et al., 2010). See Figure 1 for an overview of enterococcal biofilm formation and maturation.

## BIOFILM: NOVEL REGULATION SYSTEMS AND ANTIBIOTIC RESISTANCE TRANSFER

The search for regulatory systems central to enterococcal biofilm formation and the regulatory systems that control them has been an area of active research (Grand et al., 2019, 2020; Manias & Dunny, 2018; Zheng et al., 2017). Chilambi et al. (2020) have further extended our understanding of these complex and diverse regulatory systems by reporting on the evolution of Efs in immunocompromised patients finding that vancomycin‐resistant strains adapted during colonization and mutations accumulated that contributed to increased biofilm formation. There has been considerable effort put into defining Efs biofilm regulation in the hope of identifying a central control point that might become a novel antimicrobial drug target. While there are no outstanding candidates thus far, we are beginning to appreciate that Efs biofilm represents a complex environment that supports unexpected amounts of metabolic activity and evidence for its role in supporting horizontal gene transfer is steadily accumulating.

Information on how enterococci control biofilm has started to emerge recently with transcriptomic studies beginning to catalogue the regulatory systems involved. The report by Lim et al. (2017) was one of the first to compare the transcriptome of biofilm and planktonic grown cells, and they found an abundance of adherence‐associated proteins upregulated in the biofilm state. Significantly, they also noted that genes involved with plasmid replication and genetic exchange were also upregulated, suggesting that the biofilm could be an environment that favours horizontal gene transfer. Sirvertsen et al. (2018) proposed an enterococcus cassette chromosomal (ECC) element, which acts as a focus for genetic exchange and contributes to the large variation of accessory genes found in *Enterococcus faecium* (Efm) and may aid in adaption to new environments. Suriyanarayanan et al. (2018) applied a proteomic approach to selected clinical strains of Efs. They highlighted the central role of metabolic processes, biosynthetic processes and transport systems in biofilm grown cultures, for example, proteins associated with the shikimate kinase pathway were upregulated in a strong biofilm former, while proteins associated with secondary metabolites were downregulated. Metabolic pathway and gene ontology analyses showed higher levels of metabolic activity in a weak biofilm former. There is no clear consensus at the moment as to the most important genes involved in biofilm formation by Efs. What is becoming clearer is that the extensive genetic heterogeneity associated with this organism can be reflected in functional diversity of biofilm and a differential response to various environmental stressors.

The interplay between antibiotic resistant bacteria and biofilm has become better understood in recent years although, as recently as 2016, Stalder and Top argued that much more effort is needed to understand the physicochemical and biological mechanisms involved in gene transfer in this environment. The notion that biofilm is simply a physical barrier that impedes drug access to the cells has now largely been dispelled as overly simplistic. The area has been recently reviewed by Abe et al. (2020), who concluded that biofilm is a hot spot for horizontal gene transfer in aquatic environments. They noted the contribution of the classical mechanisms of conjugation, transduction and transformation along with a novel membrane vesicle‐mediated exchange. The latter was found to be a widespread mechanism of antibiotic resistance gene transfer although it seems to be primarily associated with Gram negative organisms.

Nagasawa et al. (2020) working with *Streptococcus mutans* biofilm showed that stress caused by sub‐MIC levels of antibiotics stimulated biofilm formation and this contributed to higher levels of horizontal gene transfer. This theme is reflected in the enterococci where a number of investigators have demonstrated a direct link between conjugation elements, biofilm formation and virulence (Bhatty et al., 2015; Parthasarathy et al., 2020; Schmitt et al., 2018). While the molecular mechanisms underlying HGT in biofilm are beginning to be elucidated there has been a shift in focus to define the impact of the process in vivo. One area that is receiving increased attention is the role of biofilm on microplastics, which have been noted as a major environmental pollutant. There is now compelling evidence that microplastics selectively enrich antibiotic resistance genes. Recent work by Wang et al. (2020) indicated that the relative abundance of integron‐integrase genes was greater on biofilm‐microplastics, potentially suggesting a higher level of horizontal gene transfer. Yang et al. (2020) reviewed current knowledge of these microbial niches, concluding that they have, so far, unknown consequences for microbial ecology and environmental processes in aquatic ecosystems. A recent report by Pazos et al. (2020) has provided the first demonstration of biofilm‐mediated association of enterococci with microplastics in a polluted ecosystem. Therefore, the need to understand the physiological processes within biofilm has never been greater, and it will only be delivered with a combination of experimental approaches encompassing molecular biology, microscopy and bacterial physiology.

## ANTIBIOTIC RESISTANCE IN ENTEROCOCCI

Enterococcus is an ancient, resilient and versatile genus able to survive under harsh conditions (Lebreton et al., 2014). This has greatly contributed to their success in the health care environment and the pathogenicity of enterococci in human disease has recently been reviewed in detail by Fiore et al. (2019). Enterococci are some of the most common healthcare associated pathogens (Hung et al., 2019; Kreidl et al., 2018) and drug‐intensive practices such as the selective decontamination of the gut, which have shown limited clinical effectiveness, have been linked to rising VRE rates. A meta‐analysis performed by DiazGranados et al. (2005) suggested that patients with bacteraemia caused by vancomycin‐resistant enterococci were more likely to die than those with vancomycin‐sensitive enterococci, and this has been backed up by other meta‐analyses. Recently, Dubler et al. (2020), examining patients with end‐stage liver disease, suggested that it is the underlying severity of the disease that predicts the outcome rather than vancomycin resistant *Enterococcus faecium* (VREfm). Nonetheless, and irrespective of the direct action of VRE during infection, these investigators recognized a central role for vancomycin resistance in driving the use of alternative antibiotics and contributing to selection pressure in favour of linezolid‐resistant isolates. Enterococci possess several intrinsic resistance phenotypes such as resistance to penicillins, aminoglycosides and cephalosporins (Hollenbeck & Rice, 2012) and are ideally placed to acquire antimicrobial resistance (AMR) genes owing to selective pressure from antimicrobial residues present in the wide range of environments they are known to inhabit (Bonten et al., 1998; Fisher & Phillips, 2009).

The most prevalent multidrug‐resistant enterococci are Efm and Efs (Arias et al., 2010; Moellering, 1992; Molechan et al., 2019). In 2008, over 50% of all identified pathogenic Efm were multidrug resistant according to a study by Hidron et al. (2008). In the same study, Hidron et al. (2008) identified that 40% of medical devices associated infections were due to vancomycin and ampicillin resistant Efm only. Efs is less commonly resistant to vancomycin and is the primary causative agent for human endocarditis (Murdoch et al., 2009). This trend has remained for more than two decades with recent surveillance reporting MDR Efm incidence rates between 25% and 59.1% (HPSC, 2018). EFs and Efm are the most clinically relevant but other infective enterococci including *E*. *durans*, *E*. *avium*, *E*. *gallinarum* and *E*. *casseliflavus* have also being identified (Ahmed & Baptiste, 2018; Gordon et al., 1992).

## ENTEROCOCCAL PATHOGENS: *E. FAECIUM* VERSUS *E. FAECALIS*

A significant factor for the rise in prominence of enterococcal infections is their growing, multidrug‐resistance (MDR) linked to their overall genome plasticity and efficacy in acquiring additional resistance determinants (Bender et al., 2016; Hegstad et al., 2010). However, the ability of Efm to benefit from a broad exchange of genetic determinants (Gao et al., 2018) contrasts with Efs, which exhibits a more limited range of genetic inputs (Leavis et al., 2006). Importantly, many of the frequent clinical isolates of Efm are resistant to four or more antibiotics including vancomycin (Arias & Murray, 2012; Zhong et al., 2019). Work by Ekwanzala et al. (2020) identified two main multilocus sequence types (ST’s), namely ST40 and ST179, which constituted 50% of isolated vancomycin resistant *Enterococcus faecalis* (VREfs). These ST’s are commonly isolated from animals, humans and the environment worldwide (Zheng et al., 2017; Zischka et al., 2015). They have been found to carry a pathogenicity island, and they display isolate specific plasmid and phage patterns. Likewise, all isolated VREfs ST40 strains were predicted to be putative human pathogens and contained considerable genomic diversity in terms of mobile genetic elements (MGEs). Of the small number of VREfm isolated in the study, the three ST’s, ST80, ST203 and ST1446, were also isolated elsewhere (Hammerum et al., 2017) and ST203 and ST80 proved to be most prevalent. As previously highlighted, the strains found in this study are part of clonal complex 17 (CC 17) and represent the majority of VREfm strains causing infections in hospital worldwide (Lee et al., 2019). Momba and co‐workers in their examination of resistome determinants of both Efs and Efm revealed a treestructure based on STs (Ekwanzala et al., 2020). They found that CRISPR‐cas systems were only found in six vancomycin resistance E. faecalis (VREfs) genomes and none of the vancomycin resistance E. faecium (VREfm) genomes contained a functional CRISPR‐cas system although the CRISPR sequences were present. Of these systems, only ST40 and ST16 VREfs contained functional Type IIA CRISPR‐cas systems. None of the ST179 VREfs contained functional CRISPR, and all of them were, therefore, dormant or orphan CRISPR.

In enterococci, the genomes forming monophylogenetic groups support previous results of speciation of enterococci based on the *gro*ESL locus (Sanderson et al., 2019; Zaheer et al., 2012). The diversity in wastewater strains may be a reflection of their origin from clinical, companion animal or agricultural sources. However, Efm and Efs are still the predominant species in wastewater likely due to the continuous input of faecal matter. The number of genes related to the mobilome increases with genome size in Efs and Efm, and this would suggest that the mobilome is a significant factor in the evolution of these bacteria in wastewater contributing to genomic expansion and diversity. There is more genetic diversity in vancomycin‐resistant Efs (Leavis et al., 2006) than Efm (Gao et al., 2018). The lack of diversity in Efm and a preponderance of AMR genes in the mobilome suggests that Efm may be more specifically adapted to clinical environments (Zhong et al., 2019).

The success of Efm and Efs evolving as multiresistant nosocomial pathogens is associated with their ability to acquire and share adaptive traits, including antimicrobial resistance genes encoded by MGEs. Mikalsen et al. (2015) investigated this mobilome in successful hospital associated genetic lineages of Efs and Efm. Although the high level of inter‐species horizontal gene transfer (HGT) must be acknowledged, the considerable species‐specificity of these MGEs indicates a separate vertical evolution of MGEs within each species, and for Efs within each ST. Genetic modelling comparing whole genome sequences suggests two clades in Efm strains (clade A and B), where clade A includes Efm associated with human infections from CC17, as opposed to clade B that contains strains of non‐hospital human origin (Galloway‐Peña et al., 2012; Lebreton et al., 2014; Palmer et al., 2012). Efs seems to be less origin and/or host‐restricted as dominant clones are shared between hospitals and the community although some CCs, including CC2, CC40 and CC87 show clear over‐representation in hospital‐associated infections (Kuch et al., 2012). Mikalsen et al. (2015) also identified a lack of Tn*916* family conjugative transposons in Efm compared to Efs, in common with most reports of this transposon family in Efs. There was also a strong correlation between the presence of Tn*916* targets and *tetM* in Efs that was not found in the Efm strains.

Efs and Efm cannot be distinguished morphologically but different genome structures have been identified between the two species (Gan et al., 2020). Another distinct difference between Efs and Efm is their interaction with bacteriophage; hence, the molecular mechanisms used by phages to infect Efs and how Efs overcomes phage infection to become resistant are important species differentiators. Chatterjee et al. (2020) identified bacterial genes essential for infection with bacteriophage VPE25. They screened a low‐complexity transposon (Tn)‐mutant library of *E*. *faecalis* OG1RF for phage resistance (Dale et al., 2015). In addition to the VPE25 receptor (Duerkop et al., 2016), transposon sequencing revealed novel Efs genes necessary for phage adsorption and optimum intracellular phage DNA replication and transcription.

When a phage infects a bacterium, it incorporates spacers into the CRISPR array within the bacterial chromosome and occasionally plasmids (Sanderson et al., 2020). The spacers are expressed as CRISPR RNAs (crRNAs) and provide a surveillance mechanism for descendant cells and guide the CRISPR/Cas system to enable cleavage of the protospacer sequence in the phage genome. The cleaved phage genomes are then cannibalized and can no longer support productive phage infection (Barrangou, 2015; Tao et al., 2018). Functional CRISPR/Cas arrays were detected in 13 Efs genomes, with all but one also containing a prophage. The lack of a functional CRISPR/Cas array was associated with multidrug resistance in Efm. Thus, genes related to phage and CRISPR/Cas arrays could potentially serve as environmental biomarkers. Genome analysis of the phage pointed to the absence of genes associated with lysogeny, suggesting that this may be more of a factor associated with Efm isolates. However, Melo et al. (2019) isolated and characterized two novel enterococcus phages, the siphovirus vB_EfaS‐Zip (Zip) and the podovirus vB_EfaP‐Max (Max) for application during wound treatment. Both phages demonstrated lytic behaviour against Efs and Efm suggesting that more work needs to be done to elucidate the interactions between phages and Efs and Efm.

## PLASMID‐BASED CONJUGATION IN ENTEROCOCCI

There are three primary conjugative systems known in enterococci—pheromone‐responsive plasmids (recently reviewed by Sterling et al., 2020), broad host range plasmids (sometimes referred to as “pheromone‐independent conjugation”) and ICE elements or conjugative transposons of which the first discovered was Tn916 in *E*. *faecalis* DS16 (Tables 1 and 2). Tn916 was originally recognized as a transposon because of its ability to insert at multiple sites on the co‐resident plasmid pAD1 (Franke & Clewell, 1981; Gawron‐Burke & Clewell, 1982). Regardless of the system, cell‐to cell contact is needed for the plasmid and the mobilized genetic elements to be transferred.

**TABLE 1:** Antibiotic resistance genes commonly found on enterococcal plasmids

Gene	Phenotype	AMR	Mobile element
*vanA*	d‐Ala‐d‐Lac ligase	Vancomycin	pS177^(a)^, pWZ1668^(b)^, pTW9^(c)^, pWZ7140^(b)^, pWZ909^(b)^, pF856^(d)^, p5753cA^(e)^, pZB18^(f)^
*vanB*	d‐alanine‐d‐lactate ligase	Vancomycin	pVEF1^(g)^, pVEF3^(h)^, pIP816^(i)^, pMG2200^(j)^, pVEF2^(g)^
*vanZ*	Teicoplanin resistance protein	Teicoplanin	pDO2^(k)^, pS177^(a)^, pWZ1668^(b)^, pTW9^(c)^, pWZ7140^(b)^, pWZ909^(b)^, pF856^(d)^, pVEF1^(g)^, pVEF3^(h)^, pIP816^(i)^, p5753cA^(e)^
*aadE*	Aminoglycoside 6‐adenylyltransferase	Streptomycin	pDO2^(k)^, pS177^(a)^, pF856^(d)^
*ermB*	rRNA adenine N‐6‐methyltransferase	Erythromycin	pS177^(a)^, pWZ1668^(b)^, pTW9^(c)^, pWZ7140^(b)^, pWZ909^(b)^, pF856^(d)^, pRUM^(l)^
*aphA*	Aminoglycoside 3′‐phosphotransferase	Kanamycin	pDO2^(k)^, pS177^(a)^, pF856^(d)^
*pRE25(m)(j)*	Aminoglycoside phosphotransferase type III	aminoglycosides	pDO2^(k)^, pRE25^(m)^
*cat*	Chloramphenicol acetyltransferase	chloramphenicol	pDO2^(k)^, pRE25^(m)^, pCPPF5^(n)^, pRUM^(l)^, pEF‐01^(o)^
*tetL*	MFS family major facilitator transporter, tetracycline: cation symporter	Tetracycline	pDO1^(k)^, pM7M2^(p)^, pAMalpha1^(q)^
*tetM*	Tetracycline resistance protein	Tetracycline	pM7M2^(p)^, pCF10^(r)^
*tetP*	Tetracycline resistance protein	Tetracycline	pDO1^(k)^
*sace*	Streptothricin acetyltransferase	Streptothricin	pDO2^(k)^

Note

Information used for table acquired using PubMed microbial gene database queries with reference to “*E*. *faecalis* and *E*.*faecium*” where appropriate. References: Halvorsen et al. (2011)^(a)^, Zhu et al. (2010)^(b)^, Unpublished NCBI Reference Sequence: NC_014726.1^(c)^, Szakacs et al. (2014)^(d)^, NCBI Reference Sequence: NC_013317.1^(e)^, NCBI Reference Sequence: NC_016967.1^(f)^, Sletvold et al. (2007)^(g)^, Sletvold et al. (2008)^(h)^, Sletvold et al. (2010)^(i)^, Zheng et al. (2009)^(j)^, Qin et al. (2012)^(k)^, Unpublished NCBI Reference Sequence: NZ_KP842560.1^(l)^, Schwarz et al. (2001)^(m)^, Liu et al. (2014)^(n)^, Liu et al. (2012)^(o)^, Li et al. (2011)^(p)^, Francia and Clewell (2002)^(q)^, Hirt et al. (2005)^(r)^.

**TABLE 2:** Enterococcal associated antimicrobial resistance containing transposons and their associations with other organisms

Transposon	Categorization	Function (genotype)	Host range
Tn916	Conjugative	Tetracycline (*TetM*)	*Enterococcus, Staphylococcus, Streptococcus, Lactococcus, Lactobacillus, Bacillus, Clostridium, Leuconostoc, Listeria, Mycoplasma, Actinobacillus, Acholeplasma, Acinetobacter, Alcaligenes, Butyrivibria, Citrobacter, Erysipelothrix, Escherichia, Fusobacterium, Granulicatella, Haemophilus, Neisseria, Pseudomonas, Thermus, Ureaplasma, Veillonella, anaerobes*
Tn917	Tn3	Erythromycin (*ErmB*)	*Enterococcus, Staphylococcus, Streptococcus, Lactococcus, Bacillus, Listeria, Paenibacillus*
Tn1546	Tn3	Vancomycin (*vanA*)	*Enterococcus, Bacillus, Staphylococcus, Oeskorvia, Streptococcus, Rhodococcus, Arcanobacterium haemolyticum, Paenibacillus*
Tn1547	composite	Vancomycin (*vanB1*)	*Enterococcus*
Tn*5281*	composite	Gentamycin (aac‐6′/aph‐2″)	*Enterococcus, Staphylococcus. aureus, Streptococcus agalactiae, Mycoplasma*

Note

Information used for table acquired using PubMed microbial gene database queries with reference to *E*. *faecalis* and *E*. *faecium* where appropriate.

**FIGURE 2:**
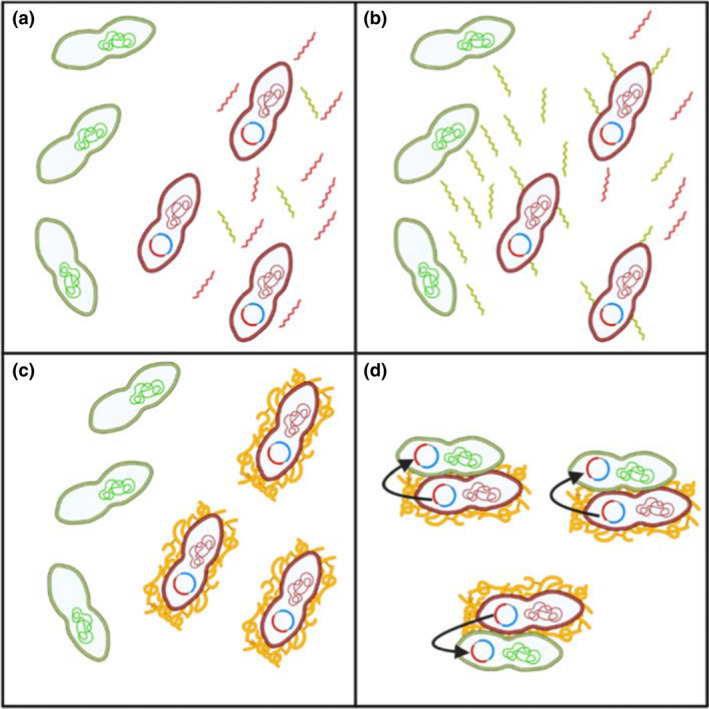
Overview of enterococcal pheromone‐based conjugation. (a) Plasmid containing enterococci secrete inhibitor sex pheromones (red extracellular peptide) into the extracellular environment to out compete against any inducer sex pheromones (green extracellular peptide). (b) When plasmid free enterococci sense a compatible plasmid containing bacteria, pheromone production is directed towards outcompeting the inhibitor production in the plasmid containing bacteria. Once a threshold has been reached and the inhibition mechanism has been overcome, binding of the pheromone occurs to the cell surface binding sites on the plasmid containing bacteria. (c) Induction of aggregation and the production of aggregation substance (yellow) occurs. (d) The plasmid containing bacteria clump together along with the plasmid free enterococci increasing surface area and allowing conjugation to occur (arrows). Created using BioRener.com with information from Dunny and Berntsson (2016)

One of the most well‐studied mechanisms of HGT in enterococci is pheromone‐responsive plasmid transfer in Efs (Dunny & Berntsson, 2016; Hirt et al., 2018; Panesso et al., 2005). The availability of proficient horizontal gene transfer, mechanisms amongst enterococci (has been reviewed in detail by Weaver, 2019) and the AMR genes associated with pheromone‐responsive plasmids are known to transfer with high efficiency (Hirt et al., 2002, 2018). The system is driven by specific short chain peptide pheromones encoded chromosomally. When these specifically bind to ‘donor’ strains, which harbour conjugative plasmids, they induce aggregation substance production (Waters et al., 2003; Waters & Dunny, 2001). These plasmid‐containing donors also produce a competing inhibitor peptide that prevents conjugation occurring between strains carrying the same plasmid. Aggregation substance is a membrane associated surface‐protein that induces clumping of donors and recipients significantly, increasing the efficiency of bacterial plasmid conjugation (Yagi et al., 1983), as seen in Figure 2. This process of HGT occurs primarily amongst Efs strains, but interspecies transfer has also been recorded with vancomycin (*vanA*) resistance being moved from Efm to Efs (Conwell et al., 2017; McCarron et al., 2019). Tetracycline resistance transfer has been demonstrated on the pheromone responsive plasmid pCF10, which has served as a model of the system for many years (Christie et al., 1987) as it has a type 4 secretion system (T4SS) (Rehman et al., 2019). These efficient pheromone responsive plasmids have shown limited replication outside the *Enterococcus* genus with transfer to *Streptococcus gordonii* being the only recognized intrageneric transmission (Mansfield et al., 2017). There is evidence for plasmids driving the evolution of specific pathogenic lineages among enterococci (Arredondo‐Alonso et al., 2020). Therefore, anything that facilitates HGT could potentially contribute to new pathogenic strains. In addition to VREs, there is evidence of commensal Efm harbouring and passing on a plasmid encoding 10 resistances. The plasmid, pEF37BA, was created from the recombination of *Erysipelothrix rhusiopathiae* chromosomal ZJ multiresistance gene cluster with the Efm's pM7M2 plasmid. This recombinant plasmid was successfully passed to another strain of Efm as wells as *Listeria welshimeri* (Morroni et al., 2019).

Conjugative plasmids, including the broad host range Inc18 group (Kohler et al., 2018), which are not pheromone dependent, are capable of transmitting antibiotic resistance across genus boundaries allowing the dissemination of antibiotic resistance to other Gram‐positive bacteria (Palmer et al., 2010). When the genome of Efs OG1RF, one of the first and most intensively studied isolates was sequenced, no foreign DNA, indicative of horizontal gene transfer (HGT) was detected (Bourgogne et al., 2008). Huo et al. (2015) identified a type II restriction modification system within OG1RF conferring a 5‐methlycytsine motif that protects it from non‐self‐DNA integration.

In contrast to the limited number of HGT events, OG1RF has been apparently incorporated over time; other enterococci can and do accumulate genetic information through a variety of methods. Conjugative transposons, which are best represented by the Tn*916* family, are mostly integrated in the chromosome. Their movement results in a non‐replicative circular intermediate that is able to transfer conjugatively, followed by stable insertion into the genome of the recipient cell. The evidence so far is limited but points to segments of DNA greater that 100kb, which appear to have been “acquired” from an unrelated source. In some cases, these ubiquitous and quite diverse elements appear to be able to conjugate (Guglielmini et al., 2011; Wozniak & Waldor, 2010). They frequently bear multiple determinants for integrase and insertion sequences, as well as genes that resemble those involved in conjugative transfer. Putative *oriT* sites, relaxase determinants and plasmid‐like conjugation genes—sometimes even similar to those found in Tn*916*—have also been identified. The presence of such determinants has given rise to the term integrative conjugative elements (ICEs), although direct demonstration of such transfer has not always been possible, with the additional presence in ICEs of genes that facilitate survival or the ability to take advantage of a new environment such as biofilms formed in vivo is common, with determinants that encode antibiotic resistance and virulence, being a good example (Tan et al., 2020).

Transposons are important genetic elements in the genomes of many enterococci, often encoding strain specific virulence and resistance phenotypes (Kristich et al., 2014). There are three main categories of enterococcal transposons: composite transposons, Tn*3* family transposons and conjugative transposons (Table 2). In terms of vancomycin phenotypes, *Van*A and *Van*B are common in Efm and Efs, but other Van genes predominate in less‐common clinically relevant species (*Van*C in *E*. *casseliflavus* and *E*. *gallinarum*) (Ahmed & Baptiste, 2018). The Tn1546 transposon carries a *VanA* gene cluster encoding resistance to vancomycin and teicoplanin (Bjørkeng et al., 2013). *Van*B is made up of subtypes (*Van*B1‐B3) (Dahl et al., 1999). The most common, the *VanB2* subtype (Hanrahan et al., 2000) is linked to a Tn5382‐like conjugative transposon. Large (90‐250kb) chromosomal elements or conjugative plasmids facilitate the intra‐ and inter‐species transfer of *vanB* (Dahl & Sundsfjord, 2003).

## ENTEROCOCCAL CONJUGATION ON SURFACES

**FIGURE 3:**
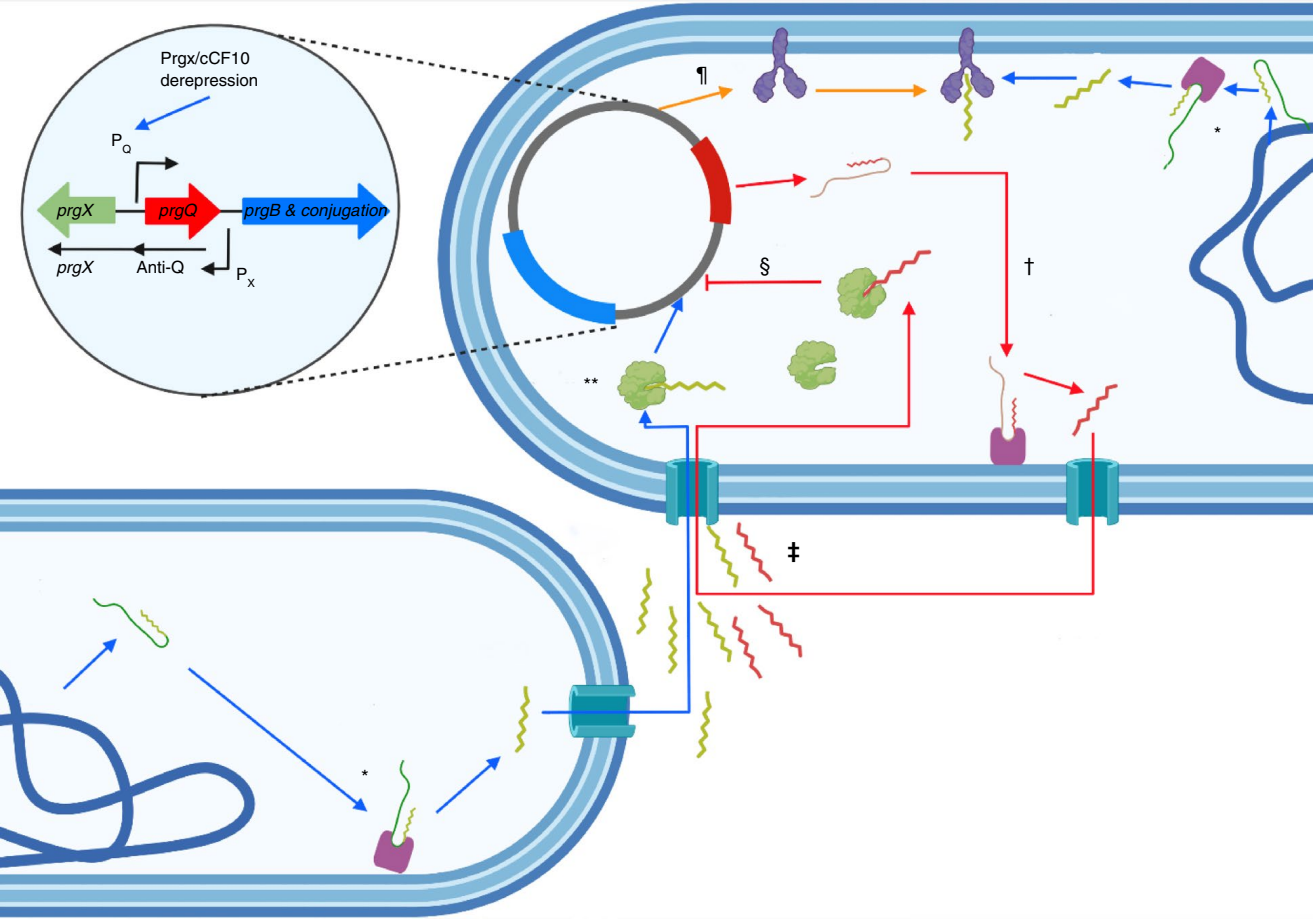
The complexities of enterococcal conjugation system using pCF10, the first fully characterised plasmid, harbouring tetracycline resistance. Enterococci secrete 8 amino acid long hydrophobic inducer pheromones expressed as part of a precursor peptide (Pro cCF10 – green peptide) encoded in the chromosomal gene *ccfa*. This peptide is cleaved by the membrane bound, enhanced expression of pheromone (Eep)*. The inducer peptide cCF10 is exported extracellularly via PptAB and is imported into the cytosol of a plasmid containing donor cell assisted via Opp/PrgZ. Enterococcal pCF10 containing cells also possess the same machinery, which is inhibited by the plasmid encoded PrgY which degrades its own cCF10 peptides to prevent auto aggregation and activation of conjugation^¶^. The RNPP regulator PrgX acts within the plasmid containing cell to repress the signalling of the P_Q_ promotor within the plasmid, preventing aggregation and activation of conjugation machinery. The plasmid pCF10 also produces an inhibitor peptide iCF10 from PrgQ^†^. This is also cleaved by Eep and exported extracellularly to act as a competitor to the inducer peptide cCF10. Both cCF10 and iCF10 are imported into the plasmid containing cell and competitively bind PrgX^‡^. Complexing of PrgX/iCF10 will further repress the P_Q_ promotor by inhibiting the access of RNA polymerase and subsequent transcription of conjugation genes^§^. When the extracellular concentration of the cleaved pheromone cCF10 reaches a threshold level, competitive binding with the inhibitor is outperformed and the pheromone is taken into the plasmid containing cell**. Complexing of PrgX/cCF10 destabilises the binding interface of PrgX to the DNA upstream of the P_Q_ promotor, derepressing RNA polymerase allowing the transcription of Asc10 aggregation protein and subsequent conjugation. Created using BioRener.com with information from Breuer et al. (2018)

While bacteria have been shown to conjugate under planktonic conditions, surface‐associated conjugation, may be a more likely mode of natural HGT due to the concentration of bacteria on solid surfaces (Aminov, 2011; Angles et al., 1993) (Table 3). In the same way, as transfer efficiencies can differ between enterococci when it comes to growth in planktonic or solid surface environments, the same could be said of transfer of each type of conjugative element in biofilm. In enterococci, the behaviour of conjugative plasmids and their transfer efficiently can vary whether the reaction occurs in a broth or on a solid surface. There are plasmids, such as pAMβ1, that transfer well on solid mating but have low transfer efficiency under broth mating conditions (Reniero et al., 1992). The plasmids such as pCF10 and pAD1 transfer with the use of a sex pheromone signalling pathway, allowing for efficient gene transfer at maximum rates of 10^−1^ transconjugants (Christie et al., 1987; Clewell et al., 1982). See Figure 3 for an overview of the pCF10 conjugation system. Enterococci use peptide pheromones to aggregate potential donor strains to facilitate HGT (Clewell, 2011; Palmer et al., 2010) and the cell‐surface protein, encoded by the *PrgB* aggregation gene is located on all pheromone‐inducible plasmids (Palmer et al., 2010). Bacteria that contain a pheromone responsive plasmid have their own pheromone production inhibited by a plasmid produced binding protein (the inhibitor – iCF10) (Clewell, 2011; Kozlowicz et al., 2006; Palmer et al., 2010). This mechanism can be overcome by the presence of un‐inhibited pheromone at a median concentration 80‐fold higher than the inhibitor, produced by a plasmid free *Enterococcus* (Hirt et al., 2002; Łysakowska et al., 2012). Once the inhibition system has been successfully out competed, downstream signalling activates the production of aggregation substance causing the clumping of the donor strain, making it receptive to conjugation (Clewell, 2011; Łysakowska et al., 2012). This allows Efs strains to conjugate with a donor strain at efficiencies up to 10^−1^ transconjugants per donor (Donelli et al., 2004; Hirt et al., 2002). Conjugation has also been previously instigated in two directional interspecies HGT of antibiotic resistance to other enterococci, staphylococci and streptococci (Gomez et al., 2011; Palmer et al., 2010).

**TABLE 3:** Horizontal gene transfer (HGT) in Enterococci under various mating conditions

Mating conditions	Mobile genetic elements utilised	Transfer efficiencies (Enterococcal recipients)	Reference
Activated sludge microcosm	pAD1, pIP1017, pIP501, Tn916	3.4 × 10^−1^, 1.1 × 10^−1^, 1.9 × 10^−7^, 9.3 × 10^−9^	Marcinek et al. (1998)
Biofilm reactor	pcF10	1:2.2 × 10^−5^	Cook et al. (2011)
Filter mating	65, 39, 6 kb plasmids	10^−1^–10^−9^	Vignaroli et al. (2011)
Cellulose filters	pAMβ1^a^	10^−4^–10^−6^	Gevers et al. (2003)
Liquid mating (static)	pcF10	10^−1^–10^−6^	Dale et al. (2015)
Solid surface mating (agar)	pSK41, pGO1, pLW1043, pSK1, pTEF1	10^−4^–10^−7^	Sobisch et al. (2019)
Liquid mating (shaken)	pCF10, pAM714, pAM378	10^−4^, 10^−3^, 10^−1^	Varahan et al. (2014)

^a^ Lactobacillus donor strain.

Bacterial biofilms have been postulated to be the location of choice for such processes (Tuson & Weibel, 2013) and although evidence is still accumulating in support of the importance of HGT in biofilms in enterococci, work by Król et al. (2013) showed widespread transferability in an *E*. *coli* biofilm. Madsen et al. (2012) argues that HGT is generally higher in biofilm communities compared to planktonic environments, but also suggests that successful introduction of plasmids into biofilm may require that plasmids are part of a biofilm at the very start of its development. Savage et al. (2013) showed that in *Staphylococcus aureus* conjugation frequencies were comparable for filter mating and biofilm (4.4 × 10^−4^ and 1.9 × 10^−4^, respectively) in comparison to planktonic culture (2.7 × 10^−8^). Van Meervenne and co‐workers in their work with *Pseudomonas putida* and *Escherichia coli* showed a plasmid transfer ratio of 1/100 in filter mating (Van Meervenne et al., 2012), conditions in comparison to biofilm data collected by Van Meerveene et al. (2014), which gave transfer ratios of between 2/100 and 1/10.

In terms of HGT in biofilm, there is a growing understanding that some important elements may be transferred in enterococcal biofilms in vivo. For instance, Abe et al. (2020) in a recent review of HGT in aquatic environments pointed to possible interconnections between HGT mechanisms and biofilms. However, it is unclear in most cases if the evidence of HGT in vivo is biofilm associated or not. There is little direct evidence of these types of studies having been carried out possibly due to the lack of appropriate experimental systems to investigate the phenomenon. Neela et al. (2009) reported that *tet*(M) was transferred from *Lactococcus garvieae* to human Efs but not to *E*. *coli*. In contrast, *Vibrio* spp. transferred *tet*(M) to *E*. *coli*, but not to Efs. These donors (*L*. *garvieae* and *Vibrio* spp.) are fish‐pathogenic bacteria and, in vivo, these organisms would form biofilms on fish intestine, where the transfer of ARGs would occur. Some conjugative plasmids facilitate biofilm development by encoding biofilm‐associated proteins. Notably, the pCF10 conjugative plasmid discussed above encodes three cell‐wall‐anchoring proteins (PrgA, PrgB and PrgC) that promote cell–cell adhesion at an early stage of biofilm formation (Bhatty et al., 2015).

While the literature on conjugative DNA transfer by enterococci and other bacteria is extensive, reports demonstrating these transfer events within biofilms are relatively scarce, reflecting the technical challenges of demonstrating the process in situ. Nonetheless, this area has been receiving increasing attention and a recent review by Abe et al. (2020) has shown that biofilm is an important site for horizontal gene transfer (HGT) in aquatic environments, and Conwell et al. (2021) have proposed a novel model to identify biofilm associated HGT using molecular imaging techniques. Abe et al. (2020) considered how HGT impacts on environmental processes and examined the major mechanisms for biofilm‐associated HGT, including the membrane vesicle‐medicated exchange. The latter process has been reviewed relatively recently by Domingues and Nielsen (2017). Prescott and Decho (2020) made the point that quorum sensing networks develop in biofilm and they are closely linked to bacterial flexibility and adaptability.

While next generation sequencing methodology is contributing extensive amounts of data on bacterial species, antibiotic resistance genes and mobile elements in biofilm, Abe et al. (2020) argue strongly for improved microscopic methods for direct visualization of biological processes within this complex matrix. It is becoming increasingly clear that the combination of experimental and bioinformatic approaches will be necessary to estimate the contribution of biofilm to emerging antibiotic resistance, and this will be an important parameter in determining how best to manage the risk from environmental hot spots.

## ENTEROCOCCAL BIOFILM—GAPS IN THE LITERATURE

While biofilm and its formation has been extensively investigated, enterococcal‐specific biofilm characteristics and formation are less well‐understood (Barnes et al., 2012). There are a limited number of reports in the literature relating to the understanding of enterococcal biofilm characteristics. Studies tend to focus on interventions to prevent or destroy enterococcal biofilm. Due to the low numbers of publications interspaced by years, there are no universally accepted standard methods for analysing biofilm in enterococci. Variations in assays of biofilm formation and characteristics for enterococci are frequently reported. This can even stretch as far as the absence of a standard biofilm formation medium and is discussed in detail, by Dunny et al. (2014), Colomer‐Winter et al. (2019), Willett et al. (2021) and briefly by Kim et al. (2020).

The consensus from the literature appears to be that our understanding of biofilm formation capability as a function of the specific characteristics of growth, substrate and biofilm promotors is based on the limited model systems available at present (Fisher & Phillips, 2009; Gilmore et al., 2014; Goh et al., 2017); with time, and additional model systems, our understanding may change. There are also distinctive biofilm formation variations based on static or laminar flow growth conditions (Garrett et al., 2008). Optimization of biofilm biomass using these characteristics may have a negative impact on the functionality of bacteria in the biofilm state, as a model of persistent antibiotic resistant infection in vivo (Nguyen et al., 2011). There is a growing consensus that bacteria modulate their biofilm to adapt to changing conditions of stress; rather than producing biofilm in large quantities as a function of their pathogenicity or growth conditions (Cambronel et al., 2020; Garrett et al., 2008; Stewart et al., 2015).

As compared to pathogenicity studies, biofilm studies are for the most part assay based and can range from a measure of biofilm formation based on cell counting alone, the ratio of cells to biomass, or dry biomass alone (Creti et al., 2006; Hufnagel et al., 2004; Kristich et al., 2004; Rosa et al., 2006). There exists a drive for insight on the workings of enterococcal biofilm, as the environment is conducive for exchange of information, especially when coupled to the knowledge of intercellular signalling pathways such as the *fsr* and Acyl‐homoserine lactone systems (McDougald et al., 2012; Parsek & Greenberg, 2000). It may be that HGT within a biofilm is inefficient as compared to laboratory methodologies (Cook et al., 2011). Understanding these processes will unlock the opportunity for a calculated approach, dealing with increasingly resistant opportunistic infections through effective treatment and preventative strategies.

In order to make relevant comparisons to pathogenic mechanisms, laboratory biofilm assays need to reflect the conditions that enterococci are exposed to during infection. Such parameters would include nutrient content, substrate composition and mechanical/chemical stress (Cambronel et al., 2020; Mohamed & Huang, 2007; Van Wamel et al., 2007). Biofilm assays that work on Gram negative, flagellated *P*. *aeruginosa*, which binds to most abiotic surfaces, are likely to be inappropriate when used in conjunction with Gram positive, non‐flagellated *E*. *faecalis*, which binds to biotic surfaces (O'Toole et al., 2000; O'Toole & Kolter, 1998).

Several biofilm formation assays, using simple apparatus, are available; however, issues exist with biofilm assays, such as the polystyrene microplate assays, which have yet to be resolved. Leuck et al. (2014) revealed that enterococcal clinical isolates, which could from biofilm on porcine heart valves produced weak and variable biofilm on polystyrene microplates. They suggested that enterococcal ex vivo biofilm formation should be performed on relevant tissue substrates. Both collagen and gelatin have been investigated as a support for biofilm formation. Gelatin has been shown to provide significant improvements on biofilm formation as compared to polystyrene and glass alone (Bukhari, 2013), whereas collagen coating has been shown to increase the polysaccharide concentration of enterococcal biofilms (Ali et al., 2020). The results obtained with gelatin coated glass align with results from Bukhari (2013), whereby substrate improvements with tissue components (collagen based) improve enterococcal biofilm formation.

There are many devices described in the literature for the study of biofilm formation. The Calgary biofilm device can only be imaged with glass bottomed microplates (Ceri et al., 1999). Coupon based biofilm apparatuses such as the drip‐flow biofilm reactor, rely on an insert that must be removed and processed, increasing chances of damage (Xu et al., 1998). In terms of biofilm, Leuck et al. (2014) stated that enterococcal *ex vivo* biofilm formation can often be weak as compared to using in vivo substrates or explanted tissue. The mechanical stresses applied to biofilm processing, such as washing with PBS carried out by Toledo‐Arana et al. (2001), applies sheer stress to biofilm cells. This is especially true when biofilm formation assays are carried out on abiotic surfaces (polystyrene) known to facilitate weak biofilm formation, as carried out by Nallapareddy et al. (2006). Therefore, there is a need to develop procedures that allow for the study of biofilm formation that limit damage to the biofilm itself.

## CONCLUSION

Bacterial pathogens efficiently pass on antimicrobial resistance genes through contact mediated HGT. Resistant members of the enterococcal family can easily form biofilm and conjugate antibiotic resistance genes, such as vancomycin determinants. There is a clear need not only for appropriate assays to study biofilms themselves but also for specific purposes such as the study of transfer of antibiotic resistance in enterococcal biofilm.

## CONFLICT OF INTEREST

The authors confirm that they have no conflict of interest.

## References

[jam15441-bib-0001] Abe, K. , Nomura, N. & Suzuki, S. (2020) Biofilms: hot spots of horizontal gene transfer (HGT) in aquatic environments, with a focus on a new HGT mechanism. FEMS Microbial Ecology, 96, fiaa031. 10.1093/femsec/fiaa031 PMC7189800 32109282

[jam15441-bib-0002] Ahmed, M.O. & Baptiste, K.E. (2018) Vancomycin‐resistant enterococci: a review of antimicrobial resistance mechanisms and perspectives of human and animal health. Microbial Drug Resistance, 24, 590–606. 29058560 10.1089/mdr.2017.0147

[jam15441-bib-0003] Ali, I.A.A. , Cheung, B.P.K. , Yau, J.Y.Y. , Matinlinna, J.P. , Lévesque, C.M. , Belibasakis, G.N. et al. (2020) The influence of substrate surface conditioning and biofilm age on the composition of *Enterococcus faecalis* biofilms. International Endodontic Journal, 53, 53–61. 31408199 10.1111/iej.13202

[jam15441-bib-0004] Aminov, R.I. (2011) Horizontal gene exchange in environmental microbiota. Frontiers in Microbiology, 2, 158. 21845185 10.3389/fmicb.2011.00158 PMC3145257

[jam15441-bib-0005] Angles, M.L. , Marshall, K.C. & Goodman, A.E. (1993) Plasmid transfer between marine bacteria in the aqueous phase and biofilms in reactor microcosms. Applied and Environment Microbiology, 59, 843–850. 10.1128/aem.59.3.843-850.1993 PMC202198 16348893

[jam15441-bib-0006] Arias, C.A. , Contreras, G.A. & Murray, B.E. (2010) Management of multidrug‐resistant enterococcal infections. Clinical Microbiology & Infection, 16, 555–562. 20569266 10.1111/j.1469-0691.2010.03214.x PMC3686902

[jam15441-bib-0007] Arias, C.A. & Murray, B.E. (2012) The rise of the *Enterococcus*: beyond vancomycin resistance. Nature Reviews Microbiology, 10, 266–278. 22421879 10.1038/nrmicro2761 PMC3621121

[jam15441-bib-0008] Arredondo‐Alonso, S. , Top, J. , McNally, A. , Puranen, S. , Pesonen, M. , Pensar, J. et al. (2020) Plasmids shaped the recent emergence of the major nosocomial pathogen *Enterococcus faecium* . MBio, 11, e03284‐19. 32047136 10.1128/mBio.03284-19 PMC7018651

[jam15441-bib-0009] Barnes, A.M. , Ballering, K.S. , Leibman, R.S. , Wells, C.L. & Dunny, G.M. (2012) *Enterococcus faecalis* produces abundant extracellular structures containing DNA in the absence of cell lysis during early biofilm formation. MBio, 3, e00193‐12. 22829679 10.1128/mBio.00193-12 PMC3413405

[jam15441-bib-0010] Barnes, A.M.T. , Dale, J.L. , Chen, Y. , Manias, D.A. , Greenwood Quaintance, K.E. , Karau, M.K. et al. (2017) *Enterococcus faecalis* readily colonizes the entire gastrointestinal tract and forms biofilms in a germ‐free mouse model. Virulence, 8, 282–296. 27562711 10.1080/21505594.2016.1208890 PMC5411234

[jam15441-bib-0011] Barrangou, R. (2015) Diversity of CRISPR‐Cas immune systems and molecular machines. Genome Biology, 16, 247. 26549499 10.1186/s13059-015-0816-9 PMC4638107

[jam15441-bib-0012] Bender, J.K. , Kalmbach, A. , Fleige, C. , Klare, I. , Fuchs, S. & Werner, G. (2016) Population structure and acquisition of the vanB resistance determinant in German clinical isolates of *Enterococcus faecium* ST192. Scientific Reports, 6, 21847. 26902259 10.1038/srep21847 PMC4763178

[jam15441-bib-0013] Bhatty, M. , Cruz, M.R. , Frank, K.L. , Laverde Gomez, J.A. , Andrade, F. , Garsin, D.A. et al. (2015) *Enterococcus faecalis* pCF10‐encoded surface proteins PrgA, PrgB (aggregation substance) and PrgC contribute to plasmid transfer, biofilm formation and virulence. Molecular Microbiology, 95, 660–677. 25431047 10.1111/mmi.12893 PMC4329047

[jam15441-bib-0014] Bjørkeng, E.K. , Hjerde, E. , Pedersen, T. , Sundsfjord, A. & Hegstad, K. (2013) ICESluvan, a 94‐Kilobase mosaic integrative conjugative element conferring interspecies transfer of VanB‐type glycopeptide resistance, a novel bacitracin resistance locus, and a toxin‐antitoxin stabilization system. Journal of Bacteriology, 195, 5381–5390. 24078615 10.1128/JB.02165-12 PMC3837959

[jam15441-bib-0015] Boles, B.R. , Thoendel, M. & Singh, P.K. (2004) Self‐generated diversity produces “insurance effects” in biofilm communities. Proceedings of the National Academy of Sciences of the United States of America, 101, 16630–16635. 15546998 10.1073/pnas.0407460101 PMC528905

[jam15441-bib-0016] Bonten, M.J. , Slaughter, S. , Ambergen, A.W. , Hayden, M.K. , van Voorhis, J. , Nathan, C. et al. (1998) The role of colonization pressure in the spread of vancomycin‐resistant enterococci: an important infection control variable. Archives of Internal Medicine, 158, 1127–1132. 9605785 10.1001/archinte.158.10.1127

[jam15441-bib-0017] Bourgogne, A. , Garsin, D.A. , Qin, X. , Singh, K.V. , Sillanpaa, J. , Yerrapragada, S. et al. (2008) Large scale variation in *Enterococcus faecalis* illustrated by the genome analysis of strain OG1RF. Genome Biology, 9, R110. 18611278 10.1186/gb-2008-9-7-r110 PMC2530867

[jam15441-bib-0018] Breuer, R.J. , Hirt, H. & Dunny, G.M. (2018) Mechanistic features of the enterococcal pCF10 Sex pheromone response and the biology of *Enterococcus faecalis* in its natural habitat. Journal of Bacteriology, 200, e00733‐17. 29437851 10.1128/JB.00733-17 PMC6018354

[jam15441-bib-0019] Bukhari, S. (2013) Biofilm formation in Enterococci and Streptococci. (Doctoral dissertation, University of Bath).

[jam15441-bib-0020] Cambronel, M. , Nilly, F. , Mesguida, O. , Boukerb, A.M. , Racine, P.‐J. , Baccouri, O. et al. (2020) Influence of catecholamines (epinephrine/norepinephrine) on biofilm formation and adhesion in pathogenic and probiotic strains of *Enterococcus faecalis* . Frontiers in Microbiology, 11, 1501. 32849320 10.3389/fmicb.2020.01501 PMC7396564

[jam15441-bib-0021] Ceri, H. , Olson, M.E. , Stremick, C. , Read, R.R. , Morck, D. & Buret, A. (1999) The Calgary Biofilm Device: new technology for rapid determination of antibiotic susceptibilities of bacterial biofilms. Journal of Clinical Microbiology, 37, 1771–1776. 10325322 10.1128/jcm.37.6.1771-1776.1999 PMC84946

[jam15441-bib-0022] Chatterjee, A. , Willett, J.L.E. , Nguyen, U.T. , Monogue, B. , Palmer, K.L. , Dunny, G.M. et al. (2020) Parallel genomics uncover novel enterococcal‐bacteriophage interactions. MBio, 11, e03120‐19. 32127456 10.1128/mBio.03120-19 PMC7064774

[jam15441-bib-0023] Chilambi, G.S. , Hinks, J. , Matysik, A. , Zhu, X. , Choo, P.Y. , Liu, X. et al. (2020) *Enterococcus faecalis* adapts to antimicrobial conjugated oligoelectrolytes by lipid rearrangement and differential expression of membrane stress response genes. Frontiers in Microbiology, 11, 155. 32117172 10.3389/fmicb.2020.00155 PMC7033496

[jam15441-bib-0024] Ch'ng, J.H. , Chong, K.K.L. , Lam, L.N. , Wong, J.J. & Kline, K.A. (2019) Biofilm‐associated infection by enterococci. Nature Reviews Microbiology, 17, 82–94. 30337708 10.1038/s41579-018-0107-z PMC13729018

[jam15441-bib-0025] Christie, P.J. , Korman, R.Z. , Zahler, S.A. , Adsit, J.C. & Dunny, G.M. (1987) Two conjugation systems associated with *Streptococcus faecalis* plasmid pCF10: identification of a conjugative transposon that transfers between *S. faecalis* and *Bacillus subtilis* . Journal of Bacteriology, 169, 2529–2536. 3034859 10.1128/jb.169.6.2529-2536.1987 PMC212112

[jam15441-bib-0026] Clewell, D.B. (2011) Tales of conjugation and sex pheromones: a plasmid and enterococcal odyssey. Mobile Genetic Elements, 1, 38–54. 22016844 10.4161/mge.1.1.15409 PMC3190283

[jam15441-bib-0027] Clewell, D.B. , Tomich, P.K. , Gawron‐Burke, M.C. , Franke, A.E. , Yagi, Y. & An, F.Y. (1982) Mapping of *Streptococcus faecalis* plasmids pAD1 and pAD2 and studies relating to transposition of Tn917. Journal of Bacteriology, 152, 1220–1230. 6292164 10.1128/jb.152.3.1220-1230.1982 PMC221629

[jam15441-bib-0028] Colomer‐Winter, C. , Lemos, J.A. & Flores‐Mireles, A.L. (2019) Biofilm assays on fibrinogen‐coated silicone catheters and 96‐well polystyrene plates. Bio‐protocol, 29, e3196. 10.21769/BioProtoc.3196 PMC6519467 31106237

[jam15441-bib-0029] Conwell, M. , Daniels, V. , Naughton, P.J. & Dooley, J.S.G. (2017) Interspecies transfer of vancomycin, erythromycin and tetracycline resistance among *Enterococcus* species recovered from agrarian sources. BMC Microbiology, 17, 19. 28100194 10.1186/s12866-017-0928-3 PMC5241992

[jam15441-bib-0030] Conwell, M. , Dooley, J.S.G. & Naughton, P.J. (2021) A novel biofilm model system to visualise conjugal transfer of vancomycin resistance by environmental enterococci. Microorganisms, 9, 789. 33918930 10.3390/microorganisms9040789 PMC8070047

[jam15441-bib-0031] Cook, L. , Chatterjee, A. , Barnes, A. , Yarwood, J. , Hu, W.S. & Dunny, G. (2011) Biofilm growth alters regulation of conjugation by a bacterial pheromone. Molecular Microbiology, 81, 1499–1510. 21843206 10.1111/j.1365-2958.2011.07786.x PMC3187857

[jam15441-bib-0032] Creti, R. , Koch, S. , Fabretti, F. , Baldassarri, L. & Huebner, J. (2006) Enterococcal colonization of the gastro‐intestinal tract: role of biofilm and environmental oligosaccharides. BMC Microbiology, 6, 60. 16834772 10.1186/1471-2180-6-60 PMC1534043

[jam15441-bib-0033] Dahl, K.H. , Simonsen, G.S. , Olsvik, Ø. & Sundsfjord, A. (1999) Heterogeneity in the vanB gene cluster of genomically diverse clinical strains of vancomycin‐resistant enterococci. Antimicrobial Agents and Chemotherapy, 43, 1105–1110. 10223921 10.1128/aac.43.5.1105 PMC89118

[jam15441-bib-0034] Dahl, K.H. & Sundsfjord, A. (2003) Transferable vanB2 Tn5382‐containing elements in fecal streptococcal strains from veal calves. Antimicrobial Agents and Chemotherapy, 47, 2579–2583. 12878522 10.1128/AAC.47.8.2579-2583.2003 PMC166075

[jam15441-bib-0035] Dale, J.L. , Cagnazzo, J. , Phan, C.Q. , Barnes, A.M. & Dunny, G.M. (2015) Multiple roles for *Enterococcus faecalis* glycosyltransferases in biofilm‐associated antibiotic resistance, cell envelope integrity, and conjugative transfer. Antimicrobial Agents and Chemotherapy, 59, 4094–4105. 25918141 10.1128/AAC.00344-15 PMC4468649

[jam15441-bib-0036] DiazGranados, C.A. , Zimmer, S.M. , Mitchel, K. & Jernigan, J.A. (2005) Comparison of mortality associated with vancomycin‐resistant and vancomycin‐susceptible enterococcal bloodstream infections: a meta‐analysis. Clinical Infectious Diseases, 41, 327–333. 16007529 10.1086/430909

[jam15441-bib-0037] Domingues, S. & Nielsen, K.M. (2017) Membrane vesicles and horizontal gene transfer in prokaryotes. Current Opinion in Microbiology, 38, 16–21. 28441577 10.1016/j.mib.2017.03.012

[jam15441-bib-0038] Donelli, G. , Paoletti, C. , Baldassarri, L. , Guaglianone, E. , Di Rosa, R. , Magi, G. et al. (2004) Sex pheromone response, clumping, and slime production in enterococcal strains isolated from occluded biliary stents. Journal of Clinical Microbiology, 42, 3419–3427. 15297477 10.1128/JCM.42.8.3419-3427.2004 PMC497634

[jam15441-bib-0039] Dubler, S. , Lenz, M. , Zimmermann, S. , Richter, D.C. , Weiss, K.H. , Mehrabi, A. et al. (2020) Does vancomycin resistance increase mortality in *Enterococcus faecium* bacteraemia after orthotopic liver transplantation? A retrospective study. Antimicrobial Resistance & Infection Control, 9, 22. 32005223 10.1186/s13756-020-0683-3 PMC6995054

[jam15441-bib-0040] Duerkop, B.A. , Huo, W. , Bhardwaj, P. , Palmer, K.L. & Hooper, L.V. (2016) Molecular basis for lytic bacteriophage resistance in Enterococci. MBio, 7, e01304‐16. 27578757 10.1128/mBio.01304-16 PMC4999554

[jam15441-bib-0041] Dunny, G.M. & Berntsson, R.P.A. (2016) Enterococcal sex pheromones: evolutionary pathways to complex, two‐signal systems. Journal of Bacteriology, 198, 1556–1562. 27021562 10.1128/JB.00128-16 PMC4959283

[jam15441-bib-0042] Dunny, G.M. , Hancock, L.E. & Shankar, N. (2014) Enterococcal biofilm structure and role in colonization and disease. In: Gilmore, M.S. , Clewell, D.B. , Ike, Y. & Shankar, N. (Eds.) Enterococcal biofilm structure and role in colonization and disease—Enterococci: from commensals to leading causes of drug resistant infection. http://www.ncbi.nlm.nih.gov/books/NBK190433/

[jam15441-bib-0043] Ekwanzala, M.D. , Dewar, J.B. , Kamika, I. & Momba, M.N.B. (2020) Comparative genomics of vancomycin‐resistant Enterococcus spp. revealed common resistome determinants from hospital wastewater to aquatic environments. Science of the Total Environment, 719, 137275. 32109727 10.1016/j.scitotenv.2020.137275

[jam15441-bib-0044] Faron, M.L. , Ledeboer, N.A. & Buchan, B.W. (2016) Resistance mechanisms, epidemiology, and approaches to screening for vancomycin‐resistant enterococcus in the health care setting. Journal of Clinical Microbiology, 54, 2436–2447. 27147728 10.1128/JCM.00211-16 PMC5035425

[jam15441-bib-0045] Fiore, E. , Van Tyne, D. & Gilmore, M.S. (2019) Pathogenicity of Enterococci. Microbiology Spectrum, 7. 10.1128/microbiolspec.gpp3-0053-2018 PMC6629438 31298205

[jam15441-bib-0046] Fisher, K. & Phillips, C. (2009) The ecology, epidemiology and virulence of *Enterococcus* . Microbiology, 155, 1749–1757. 19383684 10.1099/mic.0.026385-0

[jam15441-bib-0047] Flemming, H.C. , Wingender, J. , Szewzyk, U. , Steinberg, P. , Rice, S.A. & Kjelleberg, S. (2016) Biofilms: an emergent form of bacterial life. Nature Reviews Microbiology, 14, 563–575. 27510863 10.1038/nrmicro.2016.94

[jam15441-bib-0048] Francia, M.V. & Clewell, D.B. (2002) Amplification of the tetracycline resistance determinant of pAMα1 in *Enterococcus faecalis* requires a site‐specific recombination event involving relaxase. Journal of Bacteriology, 184, 5187–5193. 12193637 10.1128/JB.184.18.5187-5193.2002 PMC135321

[jam15441-bib-0049] Franke, A.E. & Clewell, D.B. (1981) Evidence for a chromosome‐borne resistance transposon (Tn916) in *Streptococcus faecalis* that is capable of of "conjugal" transfer in the absence of a conjugative plasmid. Journal of Bacteriology, 145, 494–502. 6257641 10.1128/jb.145.1.494-502.1981 PMC217299

[jam15441-bib-0050] Galloway‐Peña, J. , Roh, J.H. , Latorre, M. , Qin, X. & Murray, B.E. (2012) Genomic and SNP analyses demonstrate a distant separation of the hospital and community‐associated clades of *Enterococcus faecium* . PLoS One, 7, e30187. 22291916 10.1371/journal.pone.0030187 PMC3266884

[jam15441-bib-0051] Gan, Y.‐Q. , Zhang, T. , Gan, Y.‐Q. , Zhao, Z. & Zhu, B. (2020) Complete genome sequences of two *Enterococcus faecium* strains and comparative genomic analysis. Experimental and Therapeutic Medicine, 19, 2019–2028. 32104261 10.3892/etm.2020.8447 PMC7027042

[jam15441-bib-0052] Gao, W. , Howden, B.P. & Stinear, T.P. (2018) Evolution of virulence in *Enterococcus faecium*, a hospital‐adapted opportunistic pathogen. Current Opinion in Microbiology, 41, 76–82. 29227922 10.1016/j.mib.2017.11.030

[jam15441-bib-0053] Garg, S. , Mohan, B. & Taneja, N. (2017) Biofilm formation capability of enterococcal strains causing urinary tract infection vis‐a‐vis colonisation and correlation with enterococcal surface protein gene. Indian Journal of Medical Microbiology, 35, 48–52. 28303818 10.4103/ijmm.IJMM_16_102

[jam15441-bib-0054] Garrett, T.R. , Bhakoo, M. & Zhang, Z. (2008) Bacterial adhesion and biofilms on surfaces. Progress in Natural Science, 18, 1049–1056.

[jam15441-bib-0055] Gawron‐Burke, C. & Clewell, D.B. (1982) A transposon in *Streptococcus faecalis* with fertility properties. Nature, 300, 281–284. 6292725 10.1038/300281a0

[jam15441-bib-0056] Gevers, D. , Huys, G. & Swings, J. (2003) *In vitro* conjugal transfer of tetracycline resistance from Lactobacillus isolates to other Gram‐positive bacteria. FEMS Microbiology Letters, 8, 125–130. 10.1016/S0378-1097(03)00505-6 12900030

[jam15441-bib-0057] Gill, S.R. , Fouts, D.E. , Archer, G.L. , Mongodin, E.F. , DeBoy, R.T. , Ravel, J. et al. (2005) Insights on evolution of virulence and resistance from the complete genome analysis of an early methicillin‐resistant *Staphylococcus aureus* strain and a biofilm‐producing methicillin‐resistant *Staphylococcus epidermidis* strain. Journal of Bacteriology, 187, 2426–2438. 15774886 10.1128/JB.187.7.2426-2438.2005 PMC1065214

[jam15441-bib-0058] Gilmore, M.S. , Clewell, D.B. , Ike, Y. & Shankar, N. (2014) Enterococcal biofilm structure and role in colonization and disease. In Gilmore, M.S. , Clewell, D.B. , Ike, Y. and Shankar, N. (Eds.) Enterococci: from commensals to leading causes of drug resistant infection. Boston: Massachusetts Eye and Ear Infirmary. http://www.ncbi.nlm.nih.gov/books/NBK190433/ 24649510

[jam15441-bib-0059] Goh, H.M.S. , Yong, M.H.A. , Chong, K.K.L. & Kline, K.A. (2017) Model systems for the study of Enterococcal colonization and infection. Virulence, 8, 1525–1562. 28102784 10.1080/21505594.2017.1279766 PMC5810481

[jam15441-bib-0060] Gomez, J.A.L. , Hendrickx, A.P. , Willems, R.J. , Top, J. , Sava, I. , Huebner, J. et al. (2011) Intra‐and interspecies genomic transfer of the *Enterococcus faecalis* pathogenicity island. PLoS One, 29, e16720. 10.1371/journal.pone.0016720 PMC3084688 21559082

[jam15441-bib-0061] Gordon, S. , Swenson, J.M. , Hill, B.C. , Pigott, N.E. , Facklam, R.R. , Cooksey, R.C. et al. (1992) Antimicrobial susceptibility patterns of common and unusual species of enterococci causing infections in the United States. Enterococcal Study Group. Journal of Clinical Microbiology, 30, 2373–2378. 1401001 10.1128/jcm.30.9.2373-2378.1992 PMC265508

[jam15441-bib-0062] Grand, M. , Aubourg, M. , Pikis, A. , Thompson, J. , Deutscher, J. , Hartke, A. et al. (2019) Characterization of the gen locus involved in β‐1,6‐oligosaccharide utilization by *Enterococcus faecalis* . Molecular Microbiology, 112, 1744–1756. 31529727 10.1111/mmi.14390

[jam15441-bib-0063] Grand, M. , Blancato, V.S. , Espariz, M. , Deutscher, J. , Pikis, A. , Hartke, A. et al. (2020) *Enterococcus faecalis* MalR acts as a repressor of the maltose operons and additionally mediates their catabolite repression via direct interaction with seryl‐phosphorylated‐HPr. Molecular Microbiology, 113, 464–477. 31755602 10.1111/mmi.14431

[jam15441-bib-0064] Guglielmini, J. , Quintais, L. , Garcillán‐Barcia, M.P. , de la Cruz, F. & Rocha, E.P.C. (2011) The repertoire of ICE in prokaryotes underscores the unity, diversity, and ubiquity of conjugation. PLoS Genetics, 7, e1002222. 21876676 10.1371/journal.pgen.1002222 PMC3158045

[jam15441-bib-0065] Halvorsen, E.M. , Williams, J.J. , Bhimani, A.J. , Billings, E.A. & Hergenrother, P.J. (2011) Txe, an endoribonuclease of the enterococcal Axe‐Txe toxin–antitoxin system, cleaves mRNA and inhibits protein synthesis. Microbiology, 157, 387–397. 21030436 10.1099/mic.0.045492-0 PMC3090131

[jam15441-bib-0066] Hammerum, A.M. , Baig, S. , Kamel, Y. , Roer, L. , Pinholt, M. , Gumpert, H. et al. (2017) Emergence of vanA *Enterococcus faecium* in Denmark, 2005–15. Journal of Antimicrobial Chemotherapy, 72, 2184–2190. 28541565 10.1093/jac/dkx138

[jam15441-bib-0067] Hancock, L.E. & Perego, M. (2004) The *Enterococcus faecalis* fsr two‐component system controls biofilm development through production of gelatinase. Journal of Bacteriology, 186, 5629–5639. 15317767 10.1128/JB.186.17.5629-5639.2004 PMC516840

[jam15441-bib-0068] Hanrahan, J. , Hoyen, C. & Rice, L.B. (2000) Geographic distribution of a large mobile element that transfers ampicillin and vancomycin resistance between *Enterococcus faecium* strains. Antimicrobial Agents and Chemotherapy, 44, 1349–1351. 10770775 10.1128/aac.44.5.1349-1351.2000 PMC89868

[jam15441-bib-0069] Haruta, S. & Kanno, N. (2015) Survivability of microbes in natural environments and their ecological impacts. Microbes and Environments, 30, 123–125. 26094633 10.1264/jsme2.ME3002rh PMC4462920

[jam15441-bib-0070] Hegstad, K. , Mikalsen, T. , Coque, T.M. , Werner, G. & Sundsfjord, A. (2010) Mobile genetic elements and their contribution to the emergence of antimicrobial resistant *Enterococcus faecalis* and *Enterococcus faecium* . Clinical Microbiology & Infection, 16, 541–554. 20569265 10.1111/j.1469-0691.2010.03226.x

[jam15441-bib-0071] Hidron, A.I. , Edwards, J.R. , Patel, J. , Horan, T.C. , Sievert, D.M. , Pollock, D.A. et al. (2008) Antimicrobial‐resistant pathogens associated with healthcare‐associated infections: annual summary of data reported to the National Healthcare Safety Network at the Centers for Disease Control and Prevention, 2006–2007. Infection Control & Hospital Epidemiology, 29, 996–1011. 18947320 10.1086/591861

[jam15441-bib-0072] Hirt, H. , Greenwood‐Quaintance, K.E. , Karau, M.J. , Till, L.M. , Kashyap, P.C. , Patel, R. et al. (2018) *Enterococcus faecalis* sex pheromone pCF10 enhances conjugative plasmid transfer in vivo. MBio, 9, e00037‐18. 29440568 10.1128/mBio.00037-18 PMC5821081

[jam15441-bib-0073] Hirt, H. , Manias, D.A. , Bryan, E.M. , Klein, J.R. , Marklund, J.K. , Staddon, J.H. et al. (2005) Characterization of the pheromone response of the *Enterococcus faecalis* conjugative plasmid pCF10: complete sequence and comparative analysis of the transcriptional and phenotypic responses of pCF10‐containing cells to pheromone induction. Journal of Bacteriology, 187, 1044–1054. 15659682 10.1128/JB.187.3.1044-1054.2005 PMC545727

[jam15441-bib-0074] Hirt, H. , Schlievert, P.M. & Dunny, G.M. (2002) *In vivo* induction of virulence and antibiotic resistance transfer in *Enterococcus faecalis* mediated by the sex pheromone‐sensing system of pCF10. Infection and Immunity, 70, 716–723. 11796604 10.1128/iai.70.2.716-723.2002 PMC127697

[jam15441-bib-0075] Høiby, N. , Bjarnsholt, T. , Moser, C. , Bassi, G.L. , Coenye, T. , Donelli, G. et al. (2015) ESCMID guideline for the diagnosis and treatment of biofilm infections 2014. Clinical Microbiology & Infection, 21, S1–S25. 25596784 10.1016/j.cmi.2014.10.024

[jam15441-bib-0076] Hollenbeck, B.L. & Rice, L.B. (2012) Intrinsic and acquired resistance mechanisms in enterococcus. Virulence, 15, 421–433. 10.4161/viru.21282 PMC3485979 23076243

[jam15441-bib-0077] HPSC . (2018) Antimicrobial resistance in key pathogens causing invasive infections in Ireland, 2018. https://www.hpsc.ie/az/microbiologyantimicrobialresistance/europeanantimicrobialresistancesurveillancesystemearss/ears‐netdataandreports/annualreports/

[jam15441-bib-0078] Hufnagel, M. , Koch, S. , Creti, R. , Baldassarri, L. & Huebner, J. (2004) A putative sugar‐binding transcriptional regulator in a novel gene locus in *Enterococcus faecalis* contributes to production of biofilm and prolonged bacteremia in mice. Journal of Infectious Diseases, 189, 420–430. 14745699 10.1086/381150

[jam15441-bib-0079] Huijbers, P.M. , Blaak, H. , de Jong, M.C. , Graat, E.A. , Vandenbroucke‐Grauls, C.M. & de Roda Husman, A.M. (2015) Role of the environment in the transmission of antimicrobial resistance to humans: a review. Environmental Science and Technology, 49, 11993–12004. 26355462 10.1021/acs.est.5b02566

[jam15441-bib-0080] Hung, W.W. , Chen, Y.H. , Tseng, S.P. , Jao, Y.T. , Teng, L.J. & Hung, W.C. (2019) Using groEL as the target for identification of *Enterococcus faecium* clades and 7 clinically relevant Enterococcus species. Journal of Microbiology, Immunology, and Infection, 52, 255–264. 10.1016/j.jmii.2018.10.008 30473144

[jam15441-bib-0081] Huo, W. , Adams, H.M. , Zhang, M.Q. & Palmer, K.L. (2015) Genome modification in *Enterococcus faecalis* OG1RF assessed by bisulfite sequencing and single‐molecule real‐time sequencing. Journal of Bacteriology, 197, 1939–1951. 25825433 10.1128/JB.00130-15 PMC4420909

[jam15441-bib-0082] Kim, M.A. , Rosa, V. & Min, K.S. (2020) Characterization of *Enterococcus faecalis* in different culture conditions. Scientific Reports, 10, 1–8. 33318537 10.1038/s41598-020-78998-5 PMC7736865

[jam15441-bib-0083] Kohler, V. , Vaishampayan, A. & Grohmann, E. (2018) Broad‐host‐range Inc18 plasmids: occurrence, spread and transfer mechanisms. Plasmid, 99, 11–21. 29932966 10.1016/j.plasmid.2018.06.001

[jam15441-bib-0084] Kozlowicz, B.K. , Dworkin, M. & Dunny, G.M. (2006) Pheromone‐inducible conjugation in *Enterococcus faecalis*: a model for the evolution of biological complexity? International Journal of Medical Microbiology, 296, 141–147. 16503196 10.1016/j.ijmm.2006.01.040 PMC2664266

[jam15441-bib-0085] Kreidl, P. , Mayr, A. , Hinterberger, G. , Berktold, M. , Knabl, L. , Fuchs, S. et al. (2018) Outbreak report: a nosocomial outbreak of vancomycin resistant enterococci in a solid organ transplant unit. Antimicrobial Resistance & Infection Control, 7, 86. 30034798 10.1186/s13756-018-0374-5 PMC6052578

[jam15441-bib-0086] Kristich, C.J. , Li, Y.H. , Cvitkovitch, D.G. & Dunny, G.M. (2004) Esp‐independent biofilm formation by *Enterococcus faecalis* . Journal of Bacteriology, 186, 154–163. 14679235 10.1128/JB.186.1.154-163.2004 PMC365672

[jam15441-bib-0087] Kristich, C.J. , Rice, L.B. & Arias, C.A. (2014) Enterococcal infection—treatment and antibiotic resistance. https://www.ncbi.nlm.nih.gov/books/NBK190420/ 24649502

[jam15441-bib-0088] Król, J.E. , Wojtowicz, A.J. , Rogers, L.M. , Heuer, H. , Smalla, K. , Krone, S.M. et al. (2013) Invasion of *E. coli* biofilms by antibiotic resistance plasmids. Plasmid, 70, 110–119. 23558148 10.1016/j.plasmid.2013.03.003 PMC3687034

[jam15441-bib-0089] Kuch, A. , Willems, R.J.L. , Werner, G. , Coque, T.M. , Hammerum, A.M. , Sundsfjord, A. et al. (2012) Insight into antimicrobial susceptibility and population structure of contemporary human *Enterococcus faecalis* isolates from Europe. Journal of Antimicrobial Chemotherapy, 67, 551–558. 22207599 10.1093/jac/dkr544

[jam15441-bib-0090] Leavis, H.L. , Bonten, M.J. & Willems, R.J. (2006) Identification of high‐risk enterococcal clonal complexes: global dispersion and antibiotic resistance. Current Opinion in Microbiology, 9, 454–460. 16880002 10.1016/j.mib.2006.07.001

[jam15441-bib-0091] Lebreton, F. , Willems, R.J. & Gilmore, M.S. (2014) *Enterococcus* diversity, origins in nature, and gut colonization. https://www.ncbi.nlm.nih.gov/books/NBK190427/ 24649513

[jam15441-bib-0092] Lee, T. , Pang, S. , Abraham, S. & Coombs, G.W. (2019) Antimicrobial‐resistant CC17 *Enterococcus faecium*: the past, the present and the future. Journal of Global Antimicrobial Resistance, 16, 36–47. 30149193 10.1016/j.jgar.2018.08.016

[jam15441-bib-0093] Leuck, A.M. , Johnson, J.R. & Dunny, G.M. (2014) A widely used *in vitro* biofilm assay has questionable clinical significance for enterococcal endocarditis. PLoS One, 9, e107282. 25255085 10.1371/journal.pone.0107282 PMC4177788

[jam15441-bib-0094] Li, X. , Alvarez, V. , Harper, W.J. & Wang, H.H. (2011) Persistent, toxin‐antitoxin system‐independent, tetracycline resistance‐encoding plasmid from a dairy *Enterococcus faecium* isolate. Applied and Environmental Microbiology, 77, 7096–7103. 21784909 10.1128/AEM.05168-11 PMC3194845

[jam15441-bib-0095] Lim, S.Y. , Teh, C.S.J. & Thong, K.L. (2017) Biofilm‐related diseases and omics: Global transcriptional profiling of *Enterococcus faecium* reveals different gene expression patterns in the biofilm and planktonic cells. OMICS: A Journal of Integrative Biology, 21, 592–602. 29049010 10.1089/omi.2017.0119

[jam15441-bib-0096] Liu, Y. , Wang, Y. , Schwarz, S. , Wang, S. , Chen, L. , Wu, C. et al. (2014) Investigation of a multiresistance gene cfr that fails to mediate resistance to phenicols and oxazolidinones in *Enterococcus faecalis* . Journal of Antimicrobial Chemotherapy, 69, 892–898. 24272266 10.1093/jac/dkt459

[jam15441-bib-0097] Liu, Y. , Wang, Y. , Wu, C. , Shen, Z. , Schwarz, S. , Du, X.D. et al. (2012) First report of the multidrug resistance gene cfr in *Enterococcus faecalis* of animal origin. Antimicrobial Agents and Chemotherapy, 56, 1650–1654. 22203597 10.1128/AAC.06091-11 PMC3294887

[jam15441-bib-0098] Łysakowska, M.E. , Denys, A. & Sienkiewicz, M. (2012) Frequency of ace, epa and elrA genes in clinical and environmental strains of *Enterococcus faecalis* . Indian Journal of Microbiology, 52, 612–616. 24293719 10.1007/s12088-012-0285-8 PMC3516638

[jam15441-bib-0099] Madsen, J.S. , Burmølle, M. , Hansen, L.H. & Sørensen, S.J. (2012) The interconnection between biofilm formation and horizontal gene transfer. FEMS Immunology and Medical Microbiology, 65, 183–195. 22444301 10.1111/j.1574-695X.2012.00960.x

[jam15441-bib-0100] Manias, D.A. & Dunny, G.M. (2018) Expression of adhesive pili and the collagen‐binding adhesin Ace is activated by ArgR family transcription factors in *Enterococcus faecalis* . Journal of Bacteriology, 200, e00269‐18. 29986940 10.1128/JB.00269-18 PMC6112011

[jam15441-bib-0101] Mansfield, J.M. , Herrmann, P. , Jesionowski, A.M. & Vickerman, M.M. (2017) *Streptococcus gordonii* pheromone s.g.c AM373 may influence the reservoir of antibiotic resistance determinants of *Enterococcus faecalis* origin in the oral metagenome. Journal of Medical Microbiology, 66, 1635–1639. 29022550 10.1099/jmm.0.000613 PMC7001488

[jam15441-bib-0102] Marcinek, H. , Wirth, R. , Muscholl‐Silberhorn, A. & Gauer, M. (1998) *Enterococcus faecalis* gene transfer under natural conditions in municipal sewage water treatment plants. Applied and Environment Microbiology, 64, 626–632. 10.1128/aem.64.2.626-632.1998 PMC106093 9464401

[jam15441-bib-0103] McCarron, M. , Dooley, J. , Banat, I. , Arnscheidt, J. & Snelling, W. (2019) Antibiotic resistance transfer in *Enterococcus faecalis* via pheromone‐induced conjugation. Access Microbiol, 1. 10.1099/acmi.ac2019.po0573

[jam15441-bib-0104] McDougald, D. , Rice, S.A. , Barraud, N. , Steinberg, P.D. & Kjelleberg, S. (2012) Should we stay, or should we go: mechanisms and ecological consequences for biofilm dispersal. Nature Reviews Microbiology, 10, 39. 10.1038/nrmicro2695 22120588

[jam15441-bib-0105] Melo, L.D.R. , Ferreira, R. , Costa, A.R. , Oliveira, H. & Azeredo, J. (2019) Efficacy and safety assessment of two enterococci phages in an *in vitro* biofilm wound model. Scientific Reports, 9, 6643. 31040333 10.1038/s41598-019-43115-8 PMC6491613

[jam15441-bib-0106] Mikalsen, T. , Pedersen, T. , Willems, R. , Coque, T.M. , Werner, G. , Sadowy, E. et al. (2015) Investigating the mobilome in clinically important lineages of *Enterococcus faecium* and *Enterococcus faecalis* . BMC Genomics, 16, 282. 25885771 10.1186/s12864-015-1407-6 PMC4438569

[jam15441-bib-0107] Moellering, R.C. Jr (1992) Emergence of *Enterococcus* as a significant pathogen. Clinical Infectious Diseases, 14, 1173–1176. 1623072 10.1093/clinids/14.6.1173

[jam15441-bib-0108] Mohamed, J.A. & Huang, D.B. (2007) Biofilm formation by enterococci. Journal of Medical Microbiology, 56, 1581–1588. 18033823 10.1099/jmm.0.47331-0

[jam15441-bib-0109] Molechan, C. , Amoako, D.G. , Abia, A.L.K. , Somboro, A.M. , Bester, L.A. & Essack, S.Y. (2019) Molecular epidemiology of antibiotic‐resistant Enterococcus spp. from the farm‐to‐fork continuum in intensive poultry production in KwaZulu‐Natal, South Africa. Science of the Total Environment, 692, 868–878. 31539992 10.1016/j.scitotenv.2019.07.324

[jam15441-bib-0110] Monds, R.D. & O’Toole, G.A. (2009) The developmental model of microbial biofilms: ten years of a paradigm up for review. Trends in Microbiology, 17, 73–87. 19162483 10.1016/j.tim.2008.11.001

[jam15441-bib-0111] Morroni, G. , Brenciani, A. , Litta‐Mulondo, A. , Vignaroli, C. , Mangiaterra, G. , Fioriti, S. et al. (2019) Characterization of a new transferable MDR plasmid carrying the pbp5 gene from a clade B com‐mensal *Enterococcus faecium* . Journal of Antimicrobial Chemotherapy, 74, 843–850. 30649343 10.1093/jac/dky549

[jam15441-bib-0112] Murdoch, D.R. , Corey, G.R. , Hoen, B. , Miró, J.M. , Fowler, V.G. , Bayer, A.S. et al. (2009) Clinical presentation, etiology, and outcome of infective endocarditis in the 21st century: the, International Collaboration on Endocarditis‐Prospective Cohort Study. Archives of Internal Medicine, 169, 463–473. 19273776 10.1001/archinternmed.2008.603 PMC3625651

[jam15441-bib-0113] Nagasawa, R. , Sato, T. , Nomura, N. , Nakamura, T. & Senpuku, H. (2020) Potential risk of spreading resistance genes within extracellular‐DNA‐dependent biofilms of *Streptococcus mutans* in response to cell envelope stress induced by sub‐MICs of bacitracin. Applied and Environment Microbiology, 86, e00770‐20. 10.1128/AEM.00770-20 PMC7414966 32532873

[jam15441-bib-0114] Nallapareddy, S.R. , Singh, K.V. , Sillanpää, J. , Garsin, D.A. , Höök, M. , Erlandsen, S.L. et al. (2006) Endocarditis and biofilm‐associated pili of *Enterococcus faecalis* . Journal of Clinical Investigation, 116, 2799–2807. 17016560 10.1172/JCI29021 PMC1578622

[jam15441-bib-0115] Neela, F.A. , Nonaka, L. , Rahman, M.H. & Suzuki, S. (2009) Transfer of the chromosomally encoded tetracycline resistance gene tet(M) from marine bacteria to *Escherichia coli* and *Enterococcus faecalis* . World Journal of Microbiology & Biotechnology, 25, 1095–1101.

[jam15441-bib-0116] Nguyen, D. , Joshi‐Datar, A. , Lepine, F. , Bauerle, E. , Olakanmi, O. , Beer, K. et al. (2011) Active starvation responses mediate antibiotic tolerance in biofilms and nutrient‐limited bacteria. Science, 334, 982–986. 22096200 10.1126/science.1211037 PMC4046891

[jam15441-bib-0117] O’Toole, G.A. & Wong, G.C. (2016) Sensational biofilms: surface sensing in bacteria. Current Opinion in Microbiology, 30, 139–146. 26968016 10.1016/j.mib.2016.02.004 PMC4843124

[jam15441-bib-0118] O'Toole, G. , Kaplan, H.B. & Kolter, R. (2000) Biofilm formation as microbial development. Annual Review of Microbiology, 54, 49–79. 10.1146/annurev.micro.54.1.49 11018124

[jam15441-bib-0119] O'Toole, G.A. & Kolter, R. (1998) Flagellar and twitching motility are necessary for *Pseudomonas aeruginosa* biofilm development. Molecular Microbiology, 30, 295–304. 9791175 10.1046/j.1365-2958.1998.01062.x

[jam15441-bib-0120] Palmer, K.L. , Godfrey, P. , Griggs, A. , Kos, V.N. , Zucker, J. , Desjardins, C. et al. (2012). Comparative genomics of enterococci: variation in *Enterococcus faecalis*, clade structure in *E. faecium*, and defining characteristics of *E. gallinarum* and *E. casseliflavus* . mBio, 3, e00318‐11. 22354958 10.1128/mBio.00318-11 PMC3374389

[jam15441-bib-0121] Palmer, K.L. , Kos, V.N. & Gilmore, M.S. (2010) Horizontal gene transfer and the genomics of enterococcal antibiotic resistance. Current Opinion in Microbiology, 13, 632–639. 20837397 10.1016/j.mib.2010.08.004 PMC2955785

[jam15441-bib-0122] Panesso, D. , Abadía‐Patiño, L. , Vanegas, N. , Reynolds, P.E. , Courvalin, P. & Arias, C.A. (2005) Transcriptional analysis of the vanC cluster from *Enterococcus gallinarum* strains with constitutive and inducible vancomycin resistance. Antimicrobial Agents and Chemotherapy, 49, 1060–1066. 15728903 10.1128/AAC.49.3.1060-1066.2005 PMC549275

[jam15441-bib-0123] Parsek, M.R. & Greenberg, E.P. (2000) Acyl‐homoserine lactone quorum sensing in Gram negative bacteria: a signaling mechanism involved in associations with higher organisms. Proceedings of the National Academy of Sciences of the United States of America, 97, 8789–8793. 10922036 10.1073/pnas.97.16.8789 PMC34013

[jam15441-bib-0124] Parthasarathy, S. , Jordan, L.D. , Schwarting, N. , Woods, M.A. , Abdullahi, Z. , Varahan, S. et al. (2020) Involvement of chromosomally encoded homologs of the RRNPP protein family in *Enterococcus faecalis* biofilm formation and urinary tract infection pathogenesis. Journal of Bacteriology, 202, e00063‐20. 32540933 10.1128/JB.00063-20 PMC7417834

[jam15441-bib-0125] Pazos, R.S. , Suárez, J.C. & Gómez, N. (2020) Study of the plastisphere: biofilm development and presence of faecal indicator bacteria on microplastics from the Río de la Plata estuary. Ecosistemas, 29, 2069.

[jam15441-bib-0126] Pollak, S. , Omer‐Bendori, S. , Even‐Tov, E. , Lipsman, V. , Bareia, T. , Ben‐Zion, I. et al. (2016) Facultative cheating supports the coexistence of diverse quorum‐sensing alleles. Proceedings of the National Academy of Sciences of the United States of America, 113, 2152–2157. 26787913 10.1073/pnas.1520615113 PMC4776494

[jam15441-bib-0127] Prescott, R.D. & Decho, A.W. (2020) Flexibility and adaptability of quorum sensing in nature. Trends in Microbiology, 28, 436–444. 32001099 10.1016/j.tim.2019.12.004 PMC7526683

[jam15441-bib-0180] Qin, X. , Galloway‐Peña, J.R. , Sillanpaa, J. , Roh, J.H. , Nallapareddy, S. R. , Chowdhury, S. et al. (2012). Complete genome sequence of *Enterococcus faecium* strain TX16 and comparative genomic analysis of *Enterococcus faecium* genomes. BMC microbiology, 12, 135. 22769602 10.1186/1471-2180-12-135 PMC3433357

[jam15441-bib-0128] Rehman, S. , Li, Y.G. , Schmitt, A. , Lassinantti, L. , Christie, P.J. & Berntsson, R.P. (2019) Enterococcal PcfF is a ribbon‐helix‐helix protein that recruits the relaxase PcfG through binding and bending of the oriT sequence. Frontiers in Microbiology, 7, 958. 10.3389/fmicb.2019.00958 PMC6514445 31134011

[jam15441-bib-0129] Reniero, R. , Cocconcelli, P. , Bottazzi, V. & Morelli, L. (1992) High frequency of conjugation in Lactobacillus mediated by an aggregation‐promoting factor. Journal of General Microbiology, 138, 763–768.

[jam15441-bib-0130] Rochex, A. , Godon, J.J. , Bernet, N. & Escudié, R. (2008) Role of shear stress on composition, diversity and dynamics of biofilm bacterial communities. Water Research, 42, 4915–4922. 18945468 10.1016/j.watres.2008.09.015

[jam15441-bib-0131] Rosa, R. , Creti, R. , Venditti, M. , D'Amelio, R. , Arciola, C.R. , Montanaro, L. et al. (2006) Relationship between biofilm formation, the enterococcal surface protein (Esp) and gelatinase in clinical isolates of *Enterococcus faecalis* and *Enterococcus faecium* . FEMS Microbiology Letters, 256, 145–150. 16487332 10.1111/j.1574-6968.2006.00112.x

[jam15441-bib-0132] Sanderson, H. , Ortega‐Polo, R. , Zaheer, R. , Goji, N. , Amoako, K.K. , Brown, R.S. et al. (2020) Comparative genomics of multidrug‐resistant Enterococcus spp. isolated from wastewater treatment plants. BMC microbiology, 20, 20. 31980014 10.1186/s12866-019-1683-4 PMC6982392

[jam15441-bib-0133] Sanderson, H. , Ortega‐Polo, R. , McDermott, K. , Zaheer, R. , Brown, R.S. , Majury, A. et al. (2019) Comparison of biochemical and genotypic speciation methods for vancomycin‐resistant enterococci isolated from urban wastewater treatment plants. Journal of Microbiological Methods, 161, 102–110. 31071353 10.1016/j.mimet.2019.04.019

[jam15441-bib-0134] Santos‐Beneit, F. (2015) The Pho regulon: a huge regulatory network in bacteria. Frontiers in Microbiology, 6, 402. 25983732 10.3389/fmicb.2015.00402 PMC4415409

[jam15441-bib-0135] Savage, V.J. , Chopra, I. & O'Neill, A.J. (2013) *Staphylococcus aureus* biofilms promote horizontal transfer of antibiotic resistance. Antimicrobial Agents and Chemotherapy, 57, 1968–1970. 23357771 10.1128/AAC.02008-12 PMC3623343

[jam15441-bib-0136] Schmitt, A. , Jiang, K. , Camacho, M.I. , Jonna, V.R. , Hofer, A. , Westerlund, F. et al. (2018) PrgB promotes aggregation, biofilm formation, and conjugation through DNA binding and compaction. Molecular Microbiology, 109, 291–305. 29723434 10.1111/mmi.13980 PMC6158044

[jam15441-bib-0137] Schulze, A. , Mitterer, F. , Pombo, J.P. & Schild, S. (2021) Biofilms by bacterial human pathogens: Clinical relevance—development, composition and regulation—therapeutical strategies. Microbial Cell, 8, 28–56. 33553418 10.15698/mic2021.02.741 PMC7841849

[jam15441-bib-0138] Schwarz, F.V. , Perreten, V. & Teuber, M. (2001) Sequence of the 50‐kb conjugative multiresistance plasmid pRE25 from *Enterococcus faecalis* RE25. Plasmid, 46, 170–187. 11735367 10.1006/plas.2001.1544

[jam15441-bib-0139] Sivertsen, A. , Janice, J. , Pedersen, T. , Wagner, T.M. , Hegstad, J. & Hegstad, K. (2018) The Enterococcus cassette chromosome, a genomic variation enabler in Enterococci. mSphere, 3, e00402‐18. 30404935 10.1128/mSphere.00402-18 PMC6222049

[jam15441-bib-0140] Sletvold, H. , Johnsen, P.J. , Hamre, I. , Simonsen, G.S. , Sundsfjord, A. & Nielsen, K.M. (2008) Complete sequence of *Enterococcus faecium* pVEF3 and the detection of an ω‐ε‐ζ toxin–antitoxin module and an ABC transporter. Plasmid, 60, 75–85. 18511120 10.1016/j.plasmid.2008.04.002

[jam15441-bib-0141] Sletvold, H. , Johnsen, P.J. , Simonsen, G.S. , Aasnaes, B. , Sundsfjord, A. & Nielsen, K.M. (2007) Comparative DNA analysis of two vanA plasmids from *Enterococcus faecium* strains isolated from poultry and a poultry farmer in Norway. Antimicrob Agent Chemother, 51, 736–739. 10.1128/AAC.00557-06 PMC1797720 17116680

[jam15441-bib-0142] Sletvold, H. , Johnsen, P.J. , Wikmark, O.G. , Simonsen, G.S. , Sundsfjord, A. & Nielsen, K.M. (2010) Tn 1546 is part of a larger plasmid‐encoded genetic unit horizontally disseminated among clonal *Enterococcus faecium* lineages. Journal of Antimicrobial Chemotherapy, 65, 1894–1906. 20558469 10.1093/jac/dkq219 PMC2920175

[jam15441-bib-0143] Sobisch, L.Y. , Rogowski, K.M. , Fuchs, J. , Schmieder, W. , Vaishampayan, A. , Oles, P. et al. (2019) Biofilm forming antibiotic resistant Gram‐positive pathogens isolated from surfaces on the international space station. Frontiers in Microbiology, 10, 543. 30941112 10.3389/fmicb.2019.00543 PMC6433718

[jam15441-bib-0144] Sterling, A.J. , Snelling, W.J. , Naughton, P.J. , Ternan, N.G. & Dooley, J.S.G. (2020) Competent but complex communication: the phenomena of pheromone‐responsive plasmids. PLoS Path, 16, e1008310. 10.1371/journal.ppat.1008310 PMC7117660 32240270

[jam15441-bib-0145] Stewart, E.J. , Ganesan, M. , Younger, J.G. & Solomon, M.J. (2015) Artificial biofilms establish the role of matrix interactions in staphylococcal biofilm assembly and disassembly. Scientific Reports, 5, 1308. 10.1038/srep13081 PMC4536489 26272750

[jam15441-bib-0146] Suriyanarayanan, T. , Qingsong, L. , Kwang, L.T. , Mun, L.Y. , Truong, T. & Seneviratne, C.J. (2018) Quantitative proteomics of strong and weak biofilm formers of *Enterococcus faecalis* reveals novel regulators of biofilm formation. Molecular & Cellular Proteomics, 17, 643–654. 29358339 10.1074/mcp.RA117.000461 PMC5880108

[jam15441-bib-0147] Szakacs, T.A. , Kalan, L. , McConnell, M.J. , Eshaghi, A. , Shahinas, D. , McGeer, A. et al. (2014) Outbreak of vancomycin‐susceptible *Enterococcus faecium* containing the wild‐type vanA gene. Journal of Clinical Microbiology, 52, 1682–1686. 24523464 10.1128/JCM.03563-13 PMC3993680

[jam15441-bib-0148] Tan, C.A.Z. , Antypas, H. & Kline, K.A. (2020) Overcoming the challenge of establishing biofilms *in vivo*: a roadmap for Enterococci. Current Opinion in Microbiology, 53, 9–18. 32062025 10.1016/j.mib.2020.01.013

[jam15441-bib-0149] Tao, P. , Wu, X. & Rao, V. (2018) Unexpected evolutionary benefit to phages imparted by bacterial CRISPR‐Cas9. Science Advances, 4, eaar4134. 29457136 10.1126/sciadv.aar4134 PMC5812732

[jam15441-bib-0150] Tendolkar, P.M. , Baghdayan, A.S. , Gilmore, M.S. & Shankar, N. (2004) Enterococcal surface protein, Esp, enhances biofilm formation by *Enterococcus faecalis* . Infection and Immunity, 72, 6032–6039. 15385507 10.1128/IAI.72.10.6032-6039.2004 PMC517584

[jam15441-bib-0151] Thurlow, L.R. , Thomas, V.C. , Narayanan, S. , Olson, S. , Fleming, S.D. & Hancock, L.E. (2010) Gelatinase contributes to the pathogenesis of endocarditis caused by *Enterococcus faecalis* . Infection and Immunity, 78, 4936–4943. 20713628 10.1128/IAI.01118-09 PMC2976315

[jam15441-bib-0152] Toledo‐Arana, A. , Valle, J. , Solano, C. , Arrizubieta, M.J. , Cucarella, C. , Lamata, M. et al. (2001) The enterococcal surface protein, Esp, is involved in *Enterococcus faecalis* biofilm formation. Applied and Environment Microbiology, 67, 4538–4545. 10.1128/AEM.67.10.4538-4545.2001 PMC93200 11571153

[jam15441-bib-0153] Tuson, H.H. & Weibel, D.B. (2013) Bacteria‐surface interactions. Soft Matter, 14, 4368–4380. 10.1039/C3SM27705D PMC3733390 23930134

[jam15441-bib-0154] Uçkay, I. , Pires, D. , Agostinho, A. , Guanziroli, N. , Öztürk, M. , Bartolone, P. et al. (2017) Enterococci in orthopaedic infections: Who is at risk getting infected? The Journal of Infection, 75, 309–314. 28676409 10.1016/j.jinf.2017.06.008

[jam15441-bib-0155] Van Acker, H. , Van Dijck, P. & Coenye, T. (2014) Molecular mechanisms of antimicrobial tolerance and resistance in bacterial and fungal biofilms. Trends in Microbiology, 22, 326–333. 24598086 10.1016/j.tim.2014.02.001

[jam15441-bib-0156] Van Meervenne, E. , De Weirdt, R. , Van Coillie, E. , Devlieghere, F. , Herman, L. & Boon, N. (2014) Biofilm models for the food industry: hot spots for plasmid transfer? Pathogens and Disease, 70, 332–338. 24436212 10.1111/2049-632X.12134

[jam15441-bib-0157] Van Meervenne, E. , Van Coillie, E. , Kerckhof, F.M. , Devlieghere, F. , Herman, L. , De Gelder, L.S. et al. (2012) Strain‐specific transfer of antibiotic resistance from an environmental plasmid to foodborne pathogens. Journal of Biomedicine & Biotechnology, 2012, 1–8. 22791963 10.1155/2012/834598 PMC3392033

[jam15441-bib-0158] Van Wamel, W.J. , Hendrickx, A.P. , Bonten, M.J. , Top, J. , Posthuma, G. & Willems, R.J. (2007) Growth condition‐dependent Esp expression by *Enterococcus faecium* affects initial adherence and biofilm formation. Infection & Immunity, 75, 924–931. 17118984 10.1128/IAI.00941-06 PMC1828491

[jam15441-bib-0159] Varahan, S. , Harms, N. , Gilmore, M.S. , Tomich, J.M. & Hancock, L.E. (2014) An ABC transporter is required for secretion of peptide sex pheromones in *Enterococcus faecalis* . MBio, 5, e01726‐14. 25249282 10.1128/mBio.01726-14 PMC4173765

[jam15441-bib-0160] Vignaroli, C. , Zandri, G. , Aquilanti, L. , Pasquaroli, S. & Biavasco, F. (2011) Multidrug‐resistant enterococci in animal meat and faeces and co‐transfer of resistance from an *Enterococcus durans* to a human *Enterococcus faecium* . Current Microbiology, 62, 1438–1447. 21286720 10.1007/s00284-011-9880-x

[jam15441-bib-0161] Vuong, C. , Kocianova, S. , Voyich, J.M. , Yao, Y. , Fischer, E.R. , DeLeo, F.R. et al. (2004) A crucial role for exopolysaccharide modification in bacterial biofilm formation, immune evasion, and virulence. Journal of Biological Chemistry, 279, 54881–54886. 15501828 10.1074/jbc.M411374200

[jam15441-bib-0162] Wang, W. , Wang, L. , Su, J. & Xu, Z. (2020) Antibiotic susceptibility, biofilm‐forming ability, and incidence of class 1 integron of Salmonella spp., *Escherichia coli*, and *Staphylococcus aureus* isolated from various foods in a school canteen in China. Foodborne Pathogens and Disease, 17, 269–275. 31794255 10.1089/fpd.2019.2694

[jam15441-bib-0163] Waters, C.M. , Antiporta, M.H. , Murray, B.E. & Dunny, G.M. (2003) Role of the *Enterococcus faecalis* GelE protease in determination of cellular chain length, supernatant pheromone levels, and degradation of fibrin and misfolded surface proteins. Journal of Bacteriology, 185, 3613–3623. 12775699 10.1128/JB.185.12.3613-3623.2003 PMC156229

[jam15441-bib-0164] Waters, C.M. & Dunny, G.M. (2001) Analysis of functional domains of the *Enterococcus faecalis* pheromone‐induced surface protein aggregation substance. Journal of Bacteriology, 183, 5659–5667. 11544229 10.1128/JB.183.19.5659-5667.2001 PMC95458

[jam15441-bib-0165] Weaver, K.E. (2019) Enterococcal genetics. Microbiology Spectrum, 7, 2. 10.1128/microbiolspec.gpp3-0055-2018 PMC11590681 30848235

[jam15441-bib-0166] Whitchurch, C.B. , Tolker‐Nielsen, T. , Ragas, P.C. & Mattick, J.S. (2002) Extracellular DNA required for bacterial biofilm formation. Science, 295, 1487. 11859186 10.1126/science.295.5559.1487

[jam15441-bib-0167] Willett, J.L.E. , Dale, J.L. , Kwiatkowski, L.M. , Powers, J.L. , Korir, M.L. , Kohli, R. et al. (2021) Comparative biofilm assays using *Enterococcus faecalis* OG1RF identify new determinants of biofilm formation. MBio, 12, e0101121. 34126766 10.1128/mBio.01011-21 PMC8262879

[jam15441-bib-0168] Wozniak, R.A.F. & Waldor, M.K. (2010) Integrative and conjugative elements: mosaic mobile genetic elements enabling dynamic lateral gene flow. Nature Reviews Microbiology, 8, 552–563. 20601965 10.1038/nrmicro2382

[jam15441-bib-0169] Xu, K.D. , Stewart, P.S. , Xia, F. , Huang, C.T. & McFeters, G.A. (1998) Spatial physiological heterogeneity in *Pseudomonas aeruginosa* biofilm is determined by oxygen availability. Applied and Environment Microbiology, 64, 4035–4039. 10.1128/aem.64.10.4035-4039.1998 PMC106596 9758837

[jam15441-bib-0170] Yagi, Y. , Kessler, R.E. , Shaw, J.H. , Lopatin, D.E. , An, F. & Clewell, D.B. (1983) Plasmid content of *Streptococcus faecalis* strain 39–5 and identification of a pheromone (cPD1)‐induced surface antigen. Microbiology, 129, 1207–1215. 10.1099/00221287-129-4-1207 6411857

[jam15441-bib-0171] Yang, Y. , Liu, W. , Zhang, Z. , Grossart, H.‐P. & Gadd, G.M. (2020) Microplastics provide new microbial niches in aquatic environments. Applied Microbiology and Biotechnology, 104, 6501–6511. 32500269 10.1007/s00253-020-10704-x PMC7347703

[jam15441-bib-0172] Zaheer, R. , Yanke, L.J. , Church, D. , Topp, E. , Read, R.R. & McAllister, T.A. (2012) High‐throughput species identification of enterococci using pyrosequencing. Journal of Microbiological Methods, 89, 174–178. 22465481 10.1016/j.mimet.2012.03.012

[jam15441-bib-0173] Zheng, B. , Tomita, H. , Inoue, T. & Ike, Y. (2009) Isolation of VanB‐type *Enterococcus faecalis* strains from nosocomial infections: first report of the isolation and identification of the pheromone‐responsive plasmids pMG2200, encoding VanB‐type vancomycin resistance and a Bac41‐type bacteriocin, and pMG2201, encoding erythromycin resistance and cytolysin (Hly/Bac). Antimicrobial Agents and Chemotherapy, 53, 735–747. 19029325 10.1128/AAC.00754-08 PMC2630599

[jam15441-bib-0174] Zheng, J.X. , Wu, Y. , Lin, Z.W. , Pu, Z.Y. , Yao, W.M. , Chen, Z. et al. (2017) Characteristics of and virulence factors associated with biofilm formation in clinical *Enterococcus faecalis* isolates in China. Frontiers in Microbiology, 8, 2338. 29225595 10.3389/fmicb.2017.02338 PMC5705541

[jam15441-bib-0175] Zhong, Z. , Kwok, L.‐Y. , Hou, Q. , Sun, Y. , Li, W. , Zhang, H. et al. (2019) Comparative genomic analysis revealed great plasticity and environmental adaptation of the genomes of *Enterococcus faecium* . BMC Genomics, 20, 602. 31331270 10.1186/s12864-019-5975-8 PMC6647102

[jam15441-bib-0176] Zhu, W. , Murray, P.R. , Huskins, W.C. , Jernigan, J.A. , McDonald, L.C. , Clark, N.C. et al. (2010) Dissemination of an *Enterococcus* Inc18‐Like *VanA* plasmid associated with vancomycin‐resistant *Staphylococcus aureus* . Antimicrobial Agents and Chemotherapy, 54, 4314–4320. 20660665 10.1128/AAC.00185-10 PMC2944587

[jam15441-bib-0177] Zischka, M. , Künne, C.T. , Blom, J. , Wobser, D. , Sakιnç, T. , Schmidt‐Hohagen, K. et al. (2015) Comprehensive molecular, genomic and phenotypic analysis of a major clone of *Enterococcus faecalis* MLST ST40. BMC Genomics, 16, 175. 25887115 10.1186/s12864-015-1367-x PMC4374294

